# Systematic Review and Meta-analysis of the Effectiveness of Whole-school Interventions Promoting Mental Health and Preventing Risk Behaviours in Adolescence

**DOI:** 10.1007/s10964-025-02135-6

**Published:** 2025-01-27

**Authors:** Roshini Balasooriya Lekamge, Ria Jain, Jenny Sheen, Pravik Solanki, Yida Zhou, Lorena Romero, Margaret M. Barry, Leo Chen, Md Nazmul Karim, Dragan Ilic

**Affiliations:** 1 School of Public Health and Preventive Medicine, Monash University, Melbourne, VIC Australia; 2 Alfred Mental and Addiction Health, Alfred Health, Melbourne, VIC Australia; 3 St Vincent’s Hospital, Melbourne, VIC Australia; 4 Eastern Health, Melbourne, VIC Australia; 5 Department of Psychiatry, School of Translational Medicine, Monash University, Melbourne, VIC Australia; 6 Ian Potter Library, Alfred Health, Melbourne, VIC Australia; 7 Health Promotion Research Centre, University of Galway, Galway, Ireland

**Keywords:** Mental health, Mental disorders, Risk behaviours, Adolescence, Whole-school approach, Health-Promoting Schools Framework

## Abstract

Adolescence is a vulnerable period for the onset of mental disorders and risk behaviours. Based on the Health-Promoting Schools Framework, whole-school interventions offer a promising strategy in this developmentally-sensitive cohort, through championing a systems-based approach to promotion and prevention that involves the key stakeholders in an adolescent’s life. The evidence-base surrounding the effectiveness of whole-school interventions, however, remains inconclusive, partly due to the insufficient number of studies in previous meta-analyses. An updated systematic review and meta-analysis was thus conducted on the effectiveness of whole-school interventions promoting mental health and preventing risk behaviours in adolescence. From 12,897 search results, 28 studies reported in 58 publications were included. Study characteristics and implementation assessments were synthesized across studies, and quality appraisals and meta-analyses performed. Analyses identified a significant reduction in the odds of cyber-bullying by 25%, regular smoking by 31% and cyber-aggression by 37% in intervention participants compared to the control. Whole-school interventions thus offer substantial population health benefits through the reduction of these highly-prevalent issues affecting adolescents. The non-significant findings pertaining to the remaining eleven outcomes, including alcohol use, recreational drug use, anxiety, depression and positive mental health, are likely attributable to suboptimal translation of the Health-Promoting Schools Framework into practice and inadequate sensitivity to adolescents’ local developmental needs. Given the ongoing challenges faced in the implementation and evaluation of these complex interventions, this study recommends that future evaluations assess the implementation of health-promoting activities in both intervention and control conditions and actively use this implementation data in the interpretation of evaluation findings.

**Preregistration:** A pre-registered PROSPERO protocol (ID: CRD42023491619) informed this study.

## Introduction

Whole-school interventions are a systems-based approach to health promotion and prevention for youth. These interventions hold vast potential in mitigating the escalating rates of mental disorders and risk behaviours in the developmentally-sensitive period of adolescence. Previous meta-analyses of the effectiveness of whole-school interventions for youth have demonstrated inconclusive results, in part due to an insufficient number of included studies. Given the potential of these interventions, this study conducted an updated systematic review and meta-analysis of the effectiveness of whole-school interventions in promoting mental health and preventing risk behaviours among adolescent populations.

Mental disorders represent the leading cause of disease burden among youth (Kieling et al., 2024). These disorders result in 31.14 million years lost to disability, amassing over one-fifth of total years lost to disability in populations aged 5 to 24 (Kieling et al., 2024). Described as the “chronic diseases of children, adolescents and young adults”, the significant disease burden attributable to mental disorders in young people prompts concern for their lifetime impact (Kieling et al., 2024). Adolescence has been identified as a particularly vulnerable period, serving as the peak life stage for the onset of mental disorders, with 48.4% of all mental disorders having emerged by age 18 (Solmi et al., 2022).

The societal costs attributable to mental disorders are stark. The global economic burden associated with mental, neurological and substance use disorders was estimated at $8.5 trillion in 2010; a figure expected to more than double by 2030 in the absence of effective preventive strategies (Bloom et al., 2012). Mental disorders have been associated with various forms of societal disadvantage, including stigma, discrimination and social exclusion (Huggett et al., 2018); higher school dropout, incarceration and homelessness, and lower economic productivity (National Academies of Sciences, Engineering, and Medicine, 2019). This sparks the urgent need for effective promotion and prevention for youth, especially for adolescents, to offset the increasing prevalence of mental disorders and their various adverse sequelae (Patel et al., 2007).

**Fig. 1:**
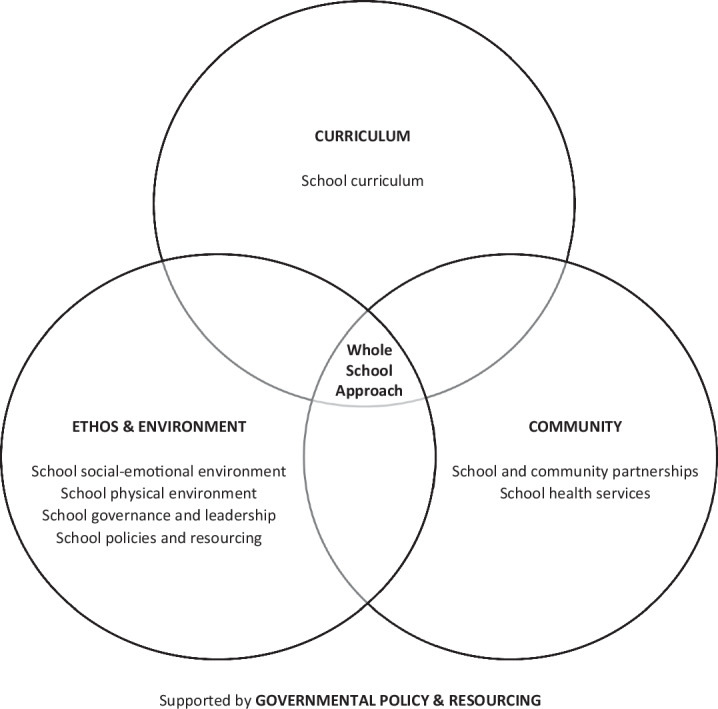
The Health-Promoting Schools Framework, adapted from the World Health Organisation and the United Nations Educational, Scientific and Cultural Organisation (World Health Organisation & the United Nations Educational, Scientific and Cultural Organisation, 2021; Langford et al., 2015; Goldberg et al., 2019)

Whole-school interventions offer a promising strategy to promote mental health and prevent risk behaviours in adolescent populations (Barry et al., 2019). Modelled on the World Health Organisation’s Health-Promoting Schools Framework (HPSF), whole-school interventions strive for change across eight domains: (i) school curriculum, (ii) school social-emotional environment, (iii) school physical environment, (iv) school governance and leadership, (v) school policies and resources, (vi) school and community partnerships, (vii) school health services, and (viii) government policies and resources (World Health Organisation & the United Nations Educational, Scientific and Cultural Organisation, 2021). In the literature, these domains are re-organized into four levels (Fig. 1), with an intervention qualifying as whole-school provided that it includes at least one programme component addressing each of the curriculum-, ethos and environment-, and community-levels (Langford et al., 2015; Goldberg et al., 2019). The argument is made that by adopting a holistic approach and by engaging the major stakeholders in an adolescent’s life, including their peers, parents and teachers, whole-school interventions offer greater potential than other forms of school-based strategies for promoting adolescent mental health and preventing risk behaviours (Barry et al., 2019; Goldberg et al., 2019; Samdal and Rowling, 2013).

**Fig. 2:**
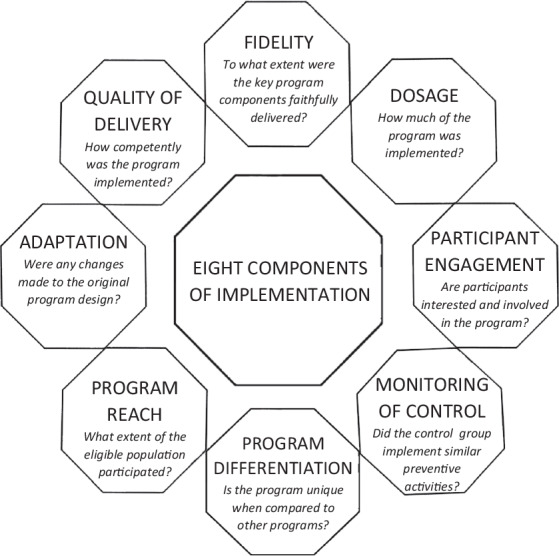
Dimensions of implementation (adapted from Durlak et al., 2016)

The current evidence examining the effectiveness of whole-school interventions promoting mental health and preventing risk behaviours in adolescents is inconclusive. A 2019 meta-analysis of whole-school interventions to social and emotional learning found small, but statistically significant improvements in social emotional adjustment, behavioural adjustment and internalizing symptoms (Goldberg et al., 2019). That review, however, was restricted to social and emotional learning interventions and did not consider the broader gamut of whole-school approaches promoting mental health and preventing risk behaviours. The most recent meta-analysis, which adopted a more comprehensive focus, was conducted in 2015 (Langford et al., 2015). The authors of that review concluded that while these interventions demonstrated evidence for reducing the odds of tobacco use and being bullied among school-aged populations, there was insufficient data to draw conclusions about mental health outcomes, violence, and drug and alcohol use (Langford et al., 2015). The consistent challenges faced in the implementation of these complex interventions has been identified as another key factor contributing to this inconclusive evidence-base (Barry et al., 2019). Here, implementation denotes the process by which an intervention is delivered and can be dissected into the eight components represented in Fig. 2 (Durlak, 2016).

## Current Study

There is inconclusive evidence on the effectiveness of whole-school interventions in promoting mental health and preventing risk behaviours in youth, partly attributable to the insufficient number of studies included in previous meta-analyses. Given the promise of whole-school interventions in mitigating the escalating rates of mental disorders and risk behaviours among adolescents, the aim of this study was to examine the effectiveness of these interventions in improving outcomes comprising positive mental health, mental disorders, substance use, bullying, aggression and mental health literacy in adolescents.

## Methods

A pre-registered PROSPERO protocol (ID: CRD42023491619) informed this study. During the title and abstract screening phase, two modifications were made to the original protocol (ID: CRD42023457678). These constituted the inclusion of three additional databases (CENTRAL, Scopus and ERIC) and restriction of the intervention inclusion criteria from universal school-based interventions to universal whole-school interventions, which are a subset of the original criterion. This study is presented in accordance with the PRISMA reporting guidelines (Page et al., 2021).

### Inclusion Criteria

Studies had to fulfil the inclusion criteria outlined in Table 1 to be eligible for this study.

**Table 1:** Inclusion criteria

Domain	Inclusion criteria
Population	Studies were included in this review if they involved participants aged 12 to 18. Where studies described participant grade rather than age, studies that involved participants in grades 7 to 12, with mean age between 12 to 18 years, were eligible for inclusion in this review.
Intervention	Similar to prior meta-analyses (Goldberg et al., 2019; Langford et al., 2015), interventions had to demonstrate a whole-school approach promoting mental health and/or preventing risk behaviours for inclusion in this review. This required at least one intervention component targeting each of the curriculum-, ethos and environment-, and community-levels of a whole-school approach. Interventions additionally had to be universal in their approach, targeting all students within a given classroom or year-level, rather than targeting subgroups at higher risk.
Comparison	Studies with an active or inactive comparison group were eligible for inclusion in this review.
Outcomes	At least one of the mental health and/or risk behaviours outlined in the WHO-UNICEF Helping Adolescents Thrive Initiative had to be reported on for inclusion in this review. These outcomes included positive mental health, mental disorders, substance use, bullying, aggression and mental health literacy (WHO, 2021; Skeen et al., 2019). Outcomes had to be quantitative in nature and could be reported by students, their teachers, parents or carers.
Type of study	Randomized controlled trials or cluster randomized controlled trials were eligible for inclusion in this review.

### Search Strategy

In total, eight databases were searched by an experienced research librarian: Ovid MEDLINE (Medical Literature Analysis and Retrieval System Online), Ovid Embase (Excerpta Medica Database), Ovid Emcare, Ovid PsycINFO (APA PsycINFO), CINAHL (Cumulative Index to Nursing and Allied Health Literature), CENTRAL (Cochrane Central Register of Controlled Trials), ERIC (Education Resources Information Centre) and Scopus. Grey literature sources identified by experts in the subject area, such as the ‘SAMHSA Evidence-Based Practice Resource Centre’ (https://www.samhsa.gov/resource-search/ebp) and ‘Blueprints for Healthy Youth Development registry of evidence-based positive youth development programmes’ (http://www.blueprintsprograms.com/programs), were likewise searched (Barry et al., 2019). Forwards and backwards citation tracking were performed. The search strategy implemented a combination of database-specific subject headings and free-text terms that encompassed the following concepts: Whole-School Approaches, Mental Health and/or Risk Behaviours, Adolescent and/or Secondary School Age Participants, and Randomized Controlled Trials. No restrictions were placed on date of publication. Studies were limited to peer-reviewed articles reported in English, with the exclusion of editorials, comments, letters, case reports, newspaper articles and reviews. The MEDLINE search strategy is included in the Supplementary Materials, with searches adjusted to fulfil the specifications of each database. All searches were undertaken from database inception until the 4^th^ of September, 2023.

### Data Extraction

**Fig. 3:**
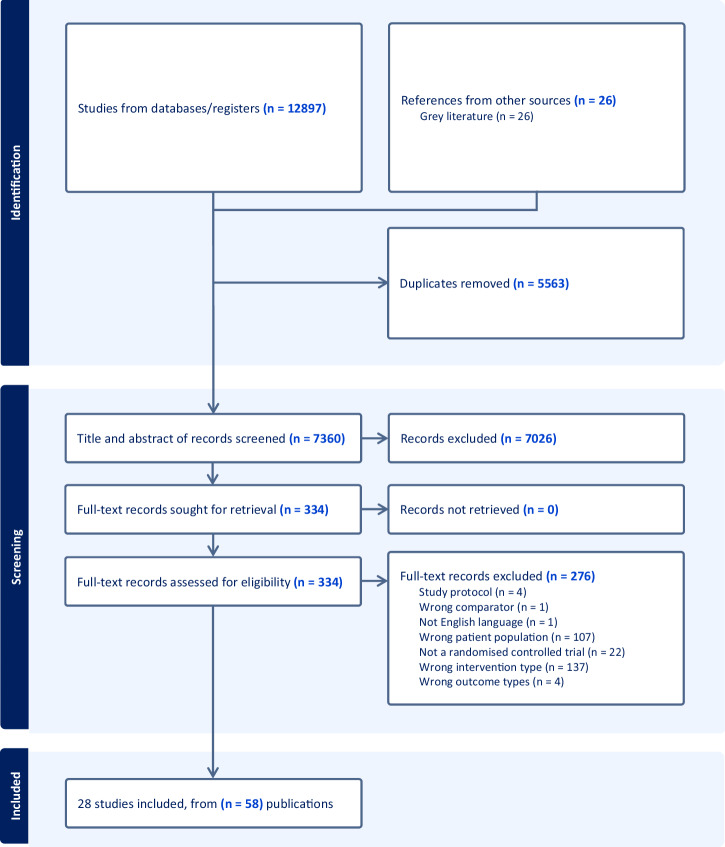
PRISMA Flowchart of Screening Process

All search results were imported into Endnote and duplicates removed. The remaining results were then imported into Covidence (Veritas Health Innovation, Melbourne, Australia), where additional duplicates were removed. Two independent reviewers (RB and either RJ, YZ, PS or JS) completed title and abstract screening, followed by full-text article screening. A third reviewer (DI) on the research team resolved discrepancies in the screening process. The screening process is summarized in Fig. 3. Two reviewers (RB and either RJ, YZ, PS or JS) independently completed data extraction in accordance with a prespecified data extraction template. The template was informed by prior meta-analyses and the theoretical concepts underlying a whole-school approach, and was piloted prior to implementation. Data extraction comprised of information on study characteristics, intervention components, implementation data, outcome measures and results on relevant outcomes. Where data was missing or unclear, corresponding authors were contacted to retrieve necessary information. While the narrative synthesis of study characteristics and mapping of intervention components against the eight domains of the Health-Promoting Schools Framework serve as the focus of a separate article (Balasooriya Lekamge et al., 2024), this article will present synthesized implementation data, quality assessments and meta-analysis results.

### Data Analysis

Two reviewers (RB and either RJ, YZ, PS or JS) independently completed risk-of-bias assessments for included studies using the Cochrane risk-of-bias tool for cluster randomized controlled trials (Eldridge et al., 2021). A third reviewer (DI) resolved disagreements in the risk-of-bias assessments. For the meta-analyses, data were grouped by the six outcome domains introduced in Table 1: positive mental health, mental disorders, substance use, bullying, aggression and mental health literacy. A further description for each outcome within these six domains is provided in Table 2. Given that outcomes were presented as either dichotomous or continuous data, the most frequent presentation in each outcome domain dictated the choice of summary estimate. All formulae for data conversions were derived from the *Campbell Collaboration Effect Size Calculator* (Wilson, 2023), the *Cochrane Collaboration Handbook for Systematic Reviews of Interventions* (Higgins et al., 2019) and the *Introduction to Meta-Analysis* textbook (Borenstein et al., 2011). In the few studies where clustering had not been accounted for in the statistical method, clustering was adjusted for using an intra-class coefficient imputed from a comparable study (Higgins et al., 2008). Where there were multiple data collection timepoints, data from the timepoint closest to post-intervention was included in the meta-analysis.

**Table 2:** Description of meta-analysis outcome domains

Domain	Outcome	Description
Substance use	Any smoking	Most studies separated outcomes for any and regular smoking. Any smoking comprises experimental or regular smoking, while regular smoking refers to ongoing, higher frequency smoking.
	Regular smoking	
	Any alcohol	Most studies separated outcomes for any and regular alcohol use. Any alcohol use comprises experimental or regular alcohol use, while regular use refers to ongoing, higher frequency use.
	Regular alcohol	
	Other substance use	Consumption of recreational substances outside of tobacco and alcohol (for example, marijuana).
Mental disorders	Anxiety	Outcomes pertaining to anxiety.
	Depression	Outcomes pertaining to depression.
	Psychological symptoms	Outcomes pertaining to psychological symptoms more broadly.
Bullying	Bullying	Outcomes pertaining to being a victim of in-person bullying.
	Cyber-bullying	Outcomes pertaining to being a victim of online bullying.
Aggression	Aggression	Outcomes pertaining to being a perpetrator of in-person aggression.
	Cyber-aggression	Outcomes pertaining to being a perpetrator of online aggression.
Positive mental health	Emotional and psychological wellbeing	Outcomes pertaining to emotional, psychological and social wellbeing (Keyes, 2013). Given that many outcome measures combine emotional and psychological wellbeing, these were presented in the one outcome category in the meta-analysis.
	Social wellbeing	
Mental health literacy	Mental health literacy	Outcomes pertaining to the understanding of how to promote and maintain mental health and wellbeing, understanding of mental disorders and available treatments, stigma related to mental disorders and help-seeking efficacy (Wei et al., 2015).

Sensitivity analyses were undertaken to interrogate the robustness of the pooled effect estimates. The first sensitivity analysis conducted was to restrict meta-analyses to include only low risk studies. Given that a high proportion of studies (25 of 28 studies) were classified as high risk for Domain 4 (detection bias) and therefore, high risk overall, a similar approach was adopted to Langford et al. (2015), where analyses were restricted by risk-of-bias domain instead of the overall risk-of-bias classification. In the second sensitivity analysis, studies were limited to those implementing single-issue interventions. Previous meta-analyses have demonstrated varying approaches, with Langford et al. (2015) stratifying all meta-analyses by intervention type (single-issue versus multiple-issue interventions), while Goldberg et al. (2019) pooled the outcomes of different intervention types together. Though the latter approach was adopted, a sensitivity analysis was conducted to investigate whether more focused, single-issue interventions produced findings that differed from the overall meta-analysis results. In the final sensitivity analysis, only studies with an inactive comparator were included. Heterogeneity was assessed using the I^2^ statistic. Heterogeneity was interpreted according to the Cochrane Handbook, where an I^2^ statistic of 0 to 40% was classified as minimal heterogeneity, 30 to 60% as moderate heterogeneity, 50 to 90% as substantial heterogeneity, and 75 to 100% as considerable heterogeneity (Deeks et al., 2023). All statistical analyses were conducted on Stata Basic Edition 18 (Stata, College Station, Texas).

## Results

### Study Characteristics

Searches returned 12,897 records, from which 28 studies reported in 58 publications met the inclusion criteria. Table 3 portrays the summary characteristics of the included studies. All studies employed cluster randomized controlled trials. The majority of studies involved students in lower secondary school grade levels, with only 5 of 28 studies involving students in Grade 10 and above. Studies were most frequently set in high-income countries, with only 5 of 28 studies set in low- and middle-income countries. Substance use prevention (10 studies) and multiple risk behaviour interventions (8 studies) were most frequently represented. The latter targeted multiple outcomes such as positive mental health, school climate, mental disorders, bullying, aggression and substance use through the single intervention. The remaining studies implemented interventions that were classified as bullying prevention (5 studies), dating violence prevention (2 studies), mindfulness promotion (1 study), depression prevention (1 study) and mental health literacy promotion (1 study). In relation to intervention duration, 8 studies spanned three-years, 10 studies 2-years, 1 study 16-months and 9 studies one-year or less. While nineteen studies described the control condition as “school-as-usual”, nine studies explicitly referenced that the control group had implemented one or more activities that coincided with the intervention’s aim. A comprehensive breakdown of study characteristics, alongside a map of how the whole-school interventions implemented by studies addressed the eight domains of the Health-Promoting Schools Framework, is found in a separate article (Balasooriya Lekamge et al., 2024).

**Table 3:** Summary characteristics of included studies

Study	Intervention type	Country	Target group	Programme duration (months)	Control	Implementation assessed	Substance use outcomes	Mental disorder outcomes	Bullying outcomes	Aggression outcomes	Positive mental health outcomes	Mental health literacy outcomes
Allara et al., 2019	Multiple risk behaviour	Italy	Grade 7 and 9	16	Inactive	X	X (MA)					
Andersen et al., 2019; Andersen et al., 2015; Bast et al., 2019; Bast et al., 2016	Smoking prevention	Denmark	Grade 7	12	Inactive	X	X (MA)					
Bond et al., 2004a, 2004b; Patton et al., 2006	Multiple risk behaviour	Australia	Grade 8	36	Inactive	X	X (MA)	X (MA)	X (MA)			
Bonell et al., 2018; Bonell et al., 2019; Bonell et al., 2020; Melendez-Torres et al., 2021; Melendez-Torres et al., 2022; Warren et al., 2019	Multiple risk behaviour	England	Grade 7	36	Inactive	X	X (MA)	X (MA)	X (MA)	X (MA)	X (MA)	
Bonnesen et al., 2023; Bonnesen et al., 2020	Multiple risk behaviour	Denmark	Grade 10	9	Inactive	X		X (MA)				
Cross et al., 2016	Cyberbullying prevention	Australia	Grade 8	24	Inactive	X			X (MA)	X (MA)		
Cross et al., 2018	Bullying prevention	Australia	Grade 8	24	Active			X (MA)	X (MA)	X (MA)		
de Vries et al., 2006 (Denmark); de Vries et al., 2003 (Denmark)	Smoking prevention	Denmark	Grade 7	36	Inactive	X	X (MA)					
de Vries et al., 2006 (Finland); de Vries et al., 2003 (Finland); Vartiainen et al., 2007	Smoking prevention	Finland	Grade 7	36	Inactive	X	X (MA)					
Dray et al., 2017; Hodder et al., 2017; Hodder et al., 2018	Multiple risk behaviour	Australia	Grade 7-10	36	Inactive	X	X (MA)	X (MA)			X (MA)	
Foshee et al., 1998; Foshee et al., 2004; Foshee et al., 2005; Foshee et al., 2014	Dating violence prevention	United States	Grade 8-9	5	Active	X				X	X	X
Gorini et al., 2014; Carreras et al., 2016	Smoking prevention	Italy	Grade 9	12	Inactive	X	X (MA)					
Hamilton et al., 2005; Hamilton et al., 2007	Smoking prevention	Australia	Grade 9	24	Active	X	X (MA)					
Hunt, 2007	Bullying prevention	Australia	Grade 7-10	2.5	Active				X (MA)	X (MA)		
Johnson et al., 2017	Mindfulness promotion	Australia	Grade 8	2.25	Inactive	X		X (MA)			X (MA)	
Kärnä et al., 2013	Bullying prevention	Finland	Grade 7-9	12	Inactive	X			X (MA)	X (MA)		
Larsen et al., 2023; Larsen et al., 2021	Multiple risk behaviour	Norway	Grade 11	36	Inactive			X (MA)			X (MA)	
Malmberg et al., 2014; Malmberg et al., 2015	Substance use prevention	The Netherlands	Grade 9	36	Inactive		X					
Perry et al., 2003; Komro et al., 2004; Bosma et al., 2005	Multiple risk behaviour	United States	Grade 7	24	Inactive	X	X			X		
Perry et al., 2009; Bate et al., 2009	Smoking prevention	India	Grade 8	24	Inactive	X	X					
Rahman et al., 1998	Mental health literacy	Pakistan	Grade 8-12	4	Inactive							X
Sawyer et al., 2010b; Sawyer et al., 2010a; Spence et al., 2014	Depression prevention	Australia	Grade 8	36	Active	X		X (MA)			X (MA)	
Schofield et al., 2003	Smoking prevention	Australia	Grade 7-8	24	Inactive	X	X					
Shinde et al., 2020; Shinde et al., 2018; Singla et al., 2021	Multiple risk behaviour	India	Grade 9	24	Active	X	X (MA)	X (MA)	X (MA)	X (MA)		
Skärstrand et al., 2014	Alcohol prevention	Sweden	Grade 6 (aged 12)	24	Active	X	X (MA)					
Stevens et al., 2000; Stevens et al., 2001	Bullying prevention	Belgium	High school students	24	Inactive	X			X (MA)	X (MA)	X (MA)	
Wen et al., 2010; Wen et al., 2007	Smoking prevention	China	Grade 7–8	24	Active	X	X (MA)					
Wolfe et al., 2009	Dating violence prevention	Canada	Grade 9	3.75	Active		X (MA)			X (MA)		

NB: Though the outcome domains have been mapped for each study, not all of these were amenable to inclusion in the respective meta-analyses. (MA) signifies that the study was included in the meta-analysis for this outcome. Reasons why studies could not be included were either because there was inadequate information to standardize outcomes for inclusion in the meta-analysis or because an inadequate number of studies existed to be meta-analyzed. Where multiple publications related to the one study, the primary publication has been listed first in the Table.

### Quality Assessments

**Fig. 4:**
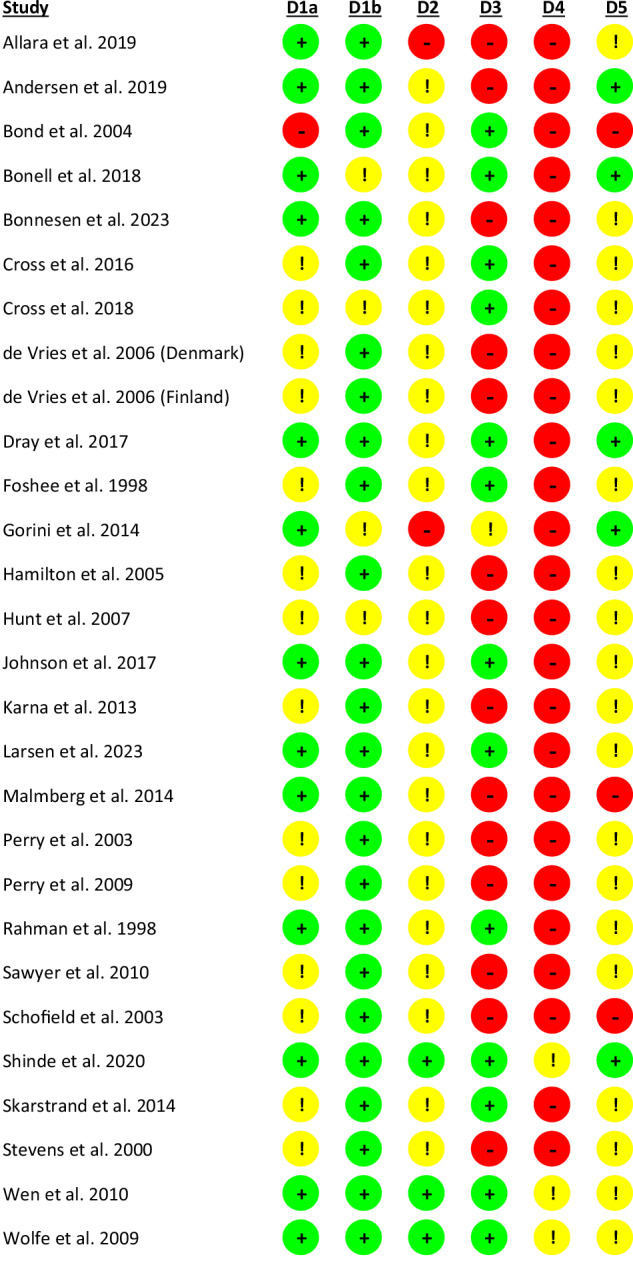
Risk-of-bias assessments for each included study. D1a and 1b assess selection bias, D2 performance bias, D3 attrition bias, D4 detection bias and D5 reporting bias. Green indicates low risk, yellow indicates unclear risk and red indicates high risk

For Domain 1a (selection bias), most studies (14 of 28 studies) were classified as unclear risk of bias with relation to random sequence generation, commonly because they described themselves as randomized trials without elaborating on the method used to generate a truly random sequence. For Domain 1b (selection bias), most studies (24 studies) were classified as low risk with relation to allocation concealment, because these studies clearly detailed the randomization of clusters at the beginning of the trial. In the assessment of Domains 1a and 1b, no marked differences emerged between the socio-demographic characteristics of the intervention and control conditions; the risk of which was likely mitigated through the use of randomization. Baseline outcomes, separated by intervention and control conditions where available, are summarized in the Supplementary Materials. The majority of studies (23 studies) were classified as unclear risk for Domain 2 (performance bias), because these studies did not explicitly reference the presence (or absence) of deviations from the study protocol. Most studies were classified as low risk (14 studies) for Domain 3 (attrition bias), including all clusters and all (or nearly all) participants within each cluster in the data analysis. Given the difficulty of blinding outcome assessors (who were often study participants) and the use of self-reporting measures, the vast majority of studies (25 studies) were classified as high risk for Domain 4 (detection bias). The majority of studies (20 studies) were classified as unclear risk for Domain 5 (reporting bias), commonly by virtue of not referencing a study protocol. Figure 4 summarizes each study’s risk for the subdomains of the risk-of-bias assessment. A detailed breakdown of risk-of-bias judgements for each study and the justification for each judgement can be found in the Supplementary Materials.

### Meta-Analyses

**Fig. 5:**
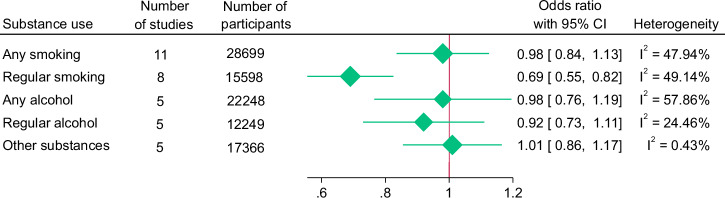
Summary forest plot for substance use outcomes

Participants receiving whole-school interventions demonstrated a statistically significant 31% decrease in the odds for regular smoking when compared to participants who did not (OR 0.69, 95% CI 0.55–0.82). There was no significant difference between participants in the intervention and control conditions for any smoking, any alcohol use, regular alcohol use or other substance use (Fig. 5). Heterogeneity varied by outcome domain, classifying as minimal for regular alcohol use; and moderate for any smoking, regular smoking, any alcohol use and other substance use. Individual forest plots for each outcome within this domain, and for each domain henceforth, can be found in the Supplementary Materials.

**Fig. 6:**
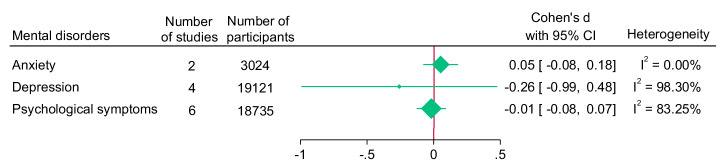
Summary forest plot for mental disorders outcomes

There was no significant difference in the effect size for anxiety, depression and psychological symptoms between participants in the intervention and control conditions (Fig. 6). Heterogeneity was minimal for anxiety, but considerable for psychological symptoms and depression.

**Fig. 7:**
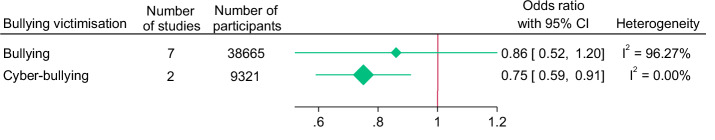
Summary forest plot for bullying outcomes

Participants receiving whole-school interventions demonstrated a statistically significant 25% reduction in the odds for being cyber-bullied when compared to those that did not (OR 0.75, 95% CI 0.59–0.91). No significant difference was found for the odds of being bullied in-person between intervention and control participants (Fig. 7). Heterogeneity was minimal for cyber-bullying, but considerable for in-person bullying.

**Fig. 8:**
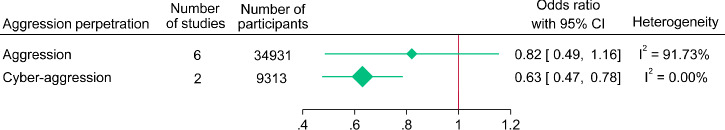
Summary forest plot for aggression outcomes

Participants receiving whole-school interventions demonstrated a statistically significant 37% decrease in the odds of perpetrating cyber-aggression as compared to those that did not (OR 0.63, 95% CI 0.47–0.78). No significant difference was found between groups for the odds of perpetrating in-person aggression (Fig. 8). Heterogeneity was minimal for cyber-aggression, but was considerable for in-person aggression.

**Fig. 9:**
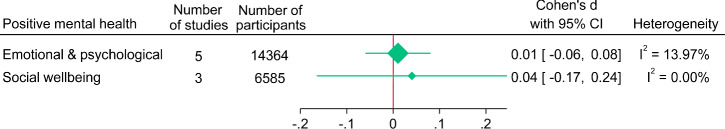
Summary forest plot for positive mental health outcomes

There was no significant difference in either indicator of positive mental health between intervention and control participants (Fig. 9). Heterogeneity was minimal for both of these outcomes.

### Outcomes not Amenable to Meta-Analysis

Two further outcomes could not be meta-analyzed due to the lack of included studies and are instead presented narratively. Shinde et al. (2020) reported on the impact of the intervention on suicide, identifying no significant difference between the intervention and control conditions in relation to the odds of suicide (OR 1.11, 95% CI 0.68–1.79). Rahman et al. (1998) examined the intervention’s impact on mental health literacy, identifying a significant improvement for participants receiving the whole-school intervention when compared to those that did not (Mean Difference 7.6, 95% CI 6.5–8.7).

### Long-Term Follow-Up

Six studies conducted additional follow-up post-intervention. Three studies that did not demonstrate a significant difference in outcomes between the intervention and control groups post-intervention, similarly did not demonstrate a significant difference at the 6- or 12-month follow-up post-intervention (Allara et al., 2019; Johnson et al., 2017; Skarstrand et al., 2014). While Bond et al. (2004a, 2004b) did not demonstrate a significant difference in outcomes post-intervention, the intervention group demonstrated a significant decrease in the odds of marked risky behaviours as compared to the control (OR 0.71, 95% CI 0.52–0.97) at follow-up two-years post-intervention (Patton et al., 2006). All other outcomes, however, remained not significantly different between the two groups. In Cross et al. (2016), the significant reduction in cyber-bullying and cyber-aggression between the intervention and control conditions post-intervention remained stable 12-months post-intervention. In Foshee et al. (2005), the intervention group demonstrated significantly less psychological abuse perpetration and moderate physical violence perpetration at post-intervention and at follow-up one, two, three and four years later.

### Sensitivity Analyses

Studies were restricted to single-issue prevention programmes (thereby excluding multiple risk behaviour interventions) to determine whether more concentrated intervention foci impacted outcomes. Where more than one study remained in the sensitivity analysis, the findings were unchanged from the overall meta-analysis results in relation to statistical significance or magnitude of the effect, except for regular smoking where substance use prevention programmes (n = 5 studies) demonstrated an increase in the magnitude of the effect (OR 0.65, 95% CI 0.46–0.84). Similarly, when restricting analyses to studies that only included an inactive comparator (thus excluding studies that employed an active comparator), no difference to the statistical significance or magnitude of overall meta-analysis results was observed.

Analyses were then restricted to studies that classified as low risk for the various subdomains of the Cochrane risk-of-bias assessments. Similar to above, outcomes had to include more than one study in the sensitivity analysis to be reported. For regular smoking, when restricted to low risk for Section 1a (n = 4), the magnitude of the effect increased (OR 0.65, 95% CI 0.35–0.95); for Section 1b (n = 6), the magnitude of the effect decreased (OR 0.74, 95% CI 0.53–0.94); for Section 3 (n = 3), the magnitude of the effect increased (OR 0.61, 95% CI 0.41–0.81) and for Section 5 (n = 2), the magnitude of the effect increased (OR 0.59, 95% CI 0.42, 0.76). For regular alcohol use, when restricted to low risk for Section 1a (n = 3), the effect became significant and demonstrated a decrease in the odds for regular alcohol use in adolescents receiving the whole-school intervention when compared to those that did not (OR 0.83, 95% 0.67–0.99). All other analyses restricted to low risk of bias did not change the statistical significance or magnitude of the effect when compared to the overall meta-analysis findings.

### Implementation Assessments

Implementation was assessed, in some capacity, by 22 of the 28 studies. Implementation fidelity and dosage were most frequently evaluated. Seven studies reported high-levels of implementation fidelity and/or dosage for the programme components monitored (Allara et al., 2019; Shinde et al., 2020; Perry et al., 2003, 2009; Karna et al., 2013; Foshee et al., 1998; Wen et al., 2010), while nine studies reported low- or mixed-levels (Bonnesen et al., 2023; Dray et al., 2017; Gorini et al., 2014; Johnson et al., 2017; Sawyer et al., 2010a, 2010b; Schofield et al., 2003; Stevens et al., 2000; Cross et al., 2016; de Vries et al., 2006). In Bonell et al. (2018), implementation fidelity was reported as high in the first and second year of the intervention, before dropping off in the third year when support from external facilitators was withdrawn. Two studies provided a breakdown of implementation fidelity between intervention schools. In Andersen et al. (2019), approximately 25% of intervention schools classified as high-implementers at both first and second follow-up, while 32% classified as low-implementers at the first follow-up, increasing to nearly 50% by the second follow-up. In Hamilton et al. (2005), approximately one-third of intervention schools classified as each of low-, moderate- and high-implementers.

The remaining six components of implementation were less frequently assessed. Staff responsiveness to the intervention was variable, trending low-moderate in two studies (Andersen et al., 2019; Bonnesen et al., 2023), moderate-high in three studies (Bonell et al., 2018; Dray et al., 2017; Sawyer et al., 2010a, 2010b), and high in one study (Johnson et al., 2017). Student responsiveness to the intervention was variable, trending low in three studies (Sawyer et al., 2010a, 2010b; de Vries et al., 2006; Wen et al., 2010), moderate in one study (Bonnesen et al., 2023) and moderate-high in two studies (Johnson et al., 2017; Andersen et al., 2019). Student reach of programme components was generally high (Bonell et al., 2018; Perry et al., 2009; Foshee et al., 1998), but parental reach was consistently low (de Vries et al., 2006; Johnson et al., 2017; Skarstrand et al., 2014). Two studies monitored the level of preventive activities implemented by the control condition (Dray et al., 2017; de Vries et al., 2006), identifying that for several intervention activities, there was no significant difference in the level of activities implemented by the intervention and control conditions, and that for certain activities, the control condition implemented significantly more activities than did the intervention condition.

Two studies examined the impact of implementation fidelity on outcomes. Hamilton et al. (2005) found that, using low-implementer schools as the reference group, adolescents at moderate-implementer schools were 0.47 times less likely (95% CI 0.20, 1.11), and high-implementer schools 0.37 times less likely (95% CI 0.15, 0.88), to smoke regularly post-intervention. Similarly, Andersen et al. (2019) found that the odds for smoking were significantly reduced for high-implementer schools when compared to the control 0.44 (95% CI 0.29, 0.65); significantly reduced albeit at a lesser magnitude for moderate-implementer schools compared to the control 0.70 (95% CI 0.54, 0.92); and low-implementer schools demonstrated no significant difference when compared to the control 0.84 (95% CI 0.63, 1.12).

## Discussion

Despite the potential of whole-school interventions in improving adolescent mental health and risk behaviour outcomes, the evidence of their effectiveness is inconclusive, in part due to the insufficient number of studies included in prior meta-analyses. This meta-analysis sought to update the evidence-base on the impact of whole-school interventions on outcomes including positive mental health, mental disorders, mental health literacy, substance use, bullying and aggression among adolescent populations.

This study identified that, of the fourteen outcomes meta-analyzed, whole-school interventions were effective in reducing cyber-bullying, cyber-aggression and regular smoking in adolescent populations. While Langford et al. (2015) demonstrated that whole-school interventions were effective in reducing the odds of being bullied among school-aged populations, the findings of this study discerned that this reduction was observed only for the odds of being bullied online, and not in-person, among adolescents. Similarly, while Langford et al. (2015) demonstrated that whole-school interventions were effective in reducing the odds of smoking among school-aged populations, the findings of this study pinpointed that this reduction was observed only for the odds of regular smoking, and not for any smoking, among adolescents. Interestingly, when restricted to low risk for selection bias, a sensitivity analysis supported the effectiveness of whole-school interventions in reducing the odds for regular alcohol use, whereas the non-significant impact on any alcohol use remained unchanged. This suggests that whole-school interventions are effective in relation to the secondary prevention of regular substance use, whereas the primary prevention of non-regular substance use (or experimental use) in adolescents presents as more reticent to change. This study further highlights an additional benefit that had not been identified by Langford et al. (2015), where whole-school interventions were found to be effective in reducing the odds of perpetrating cyber-aggression among adolescents. Given that the whole-school interventions evaluated were universal in nature, involving all adolescents rather than a select high-risk category, the population health benefits offered in relation to these outcomes is large. In an estimated adolescent population of 1.3 billion globally (UNICEF, 2024), the prevalence of cyber-bullying is estimated at up to 57.5% (Zhu et al., 2021), smoking at 19.3% (Nazir et al., 2019) and cyber-aggression at up to 46.3% (Zhu et al., 2021). The significant reduction identified by this review in the odds of cyber-bullying, regular smoking and cyber-aggression by 25%, 31 and 37% respectively, highlights that whole-school interventions can confer substantial benefit not only through the reduction of these highly-prevalent public health issues afflicting adolescents, but further, through averting their various adverse sequelae.

The analyses identified that whole-school interventions were ineffective in relation to the remaining eleven outcomes. This is a surprising finding, given that the HPSF offers a robust theoretical framework, which champions a holistic approach to promotion and prevention and involves the major stakeholders in an adolescent’s life. In unravelling this dichotomy, a key issue emerges in how the HPSF has been translated into practice. To qualify as a whole-school intervention in the literature, studies do not have to reference that the HPSF informed their intervention design. As a consequence, 75% of the studies included in this meta-analysis did not reference the HPSF (Balasooriya Lekamge et al., 2024). Instead, the literature has operationalised the definition of a whole-school intervention to be one that includes at least one programme component addressing each of the curriculum-, ethos and environment-, and community-levels outlined in Fig. 1 (Goldberg et al., 2019; Langford et al., 2015). This operationalisation of whole-school interventions in practice does not clearly align with what the World Health Organisation have advocated for through the HPSF, and as such, may not fully represent or realize the potential of the Framework. For example, a corresponding review mapped how frequently the whole-school interventions implemented by the included studies addressed the eight domains of the HPSF, identifying significant variability with which the domains were addressed (Balasooriya Lekamge et al., 2024). While 100% of interventions addressed the school curriculum domain, only 46% addressed the school physical environment domain, 29% the school health services domain, and 7% referenced the government policies and resources domain. Furthermore, studies occasionally demonstrated passive strategies to target particular domains, with posters serving as the most commonly-implemented strategy in the school physical environment domain. This begs a fundamental question: *how whole-school are whole-school interventions* (Balasooriya Lekamge et al., 2024)? Suboptimal application (or for many of the included studies, *no application*) of the HPSF may explain the dissonance between what is a theoretically robust construct and the non-significant findings observed. These findings urge reconsideration of whether interventions must explicitly be based on the HPSF to qualify as a whole-school intervention, and identify three foci for future research (Balasooriya Lekamge et al., 2024). Firstly, which of the eight domains of the HPSF are critical to the success of whole-school interventions? Secondly, does addressing more domains translate to greater impact? Thirdly, what is the relative effectiveness of intervention strategies within each domain, so that the most effective can be prioritized? Deepening our understanding of these research foci can facilitate optimal translation of the HPSF into practice to successfully promote mental health and prevent risk behaviours among adolescent populations.

In promoting the optimal application of the HPSF in practice, it is similarly critical to ensure that studies of whole-school interventions are responsive to the developmental needs of adolescents. This is particularly pertinent given that almost one-third of the included studies traversed multiple grade levels (Balasooriya Lekamge et al., 2024); for instance, a three-year study may target students as they progress from grade 7 through to grade 9. While this meta-analysis had intended to conduct a subgroup analysis to explore whether differential intervention effects were observed for adolescents in different grade levels, an insufficient number of studies for each meta-analysis precluded this analysis (Higgins et al., 2019). This developmental perspective, however, is equally crucial to consider from the lens of intervention design. In their two-year study, Cross et al. (2018) highlighted that participants found the intervention content less relevant in grade 9 than in grade 8, which corresponded with the intervention being evaluated as effective in grade 8, but not in grade 9. Sawyer et al. (2010a, 2010b) similarly identified that teacher ratings of the developmental-appropriateness of the intervention content was, on average, 59% over the three-year period, while adolescents rated their likelihood of recommending the programme to a friend as 32%. These authors subsequently cautioned against simply “aging up” intervention strategies from one grade to another, instead advocating for strategies to be tailored to each grade’s unique needs, as informed by the developmental literature (Cross et al., 2018). It is similarly imperative to highlight that the developmental needs of adolescents will be contingent on the local context. For example, reviews have identified that adolescents from socioeconomically disadvantaged settings are two to three times more likely to develop mental health problems (Reiss, 2013), with economic stress, chaos in the home environment and community violence serving as causal factors unique to this cohort (Devenish et al., 2017). Thus, in addition to grounding the design of whole-school interventions and their evaluations in the developmental literature, it is equally crucial to involve adolescents in the co-creation of intervention strategies to ensure that strategies truly capture their *local* development needs, preferences and interests.

This study further prompts reflection on the challenges faced in the evaluation of complex public health initiatives. While randomized controlled trials typically represent the most robust trial design for interventional studies, their use in the evaluation of whole-school interventions is contentious. While it is acknowledged that randomized controlled trials are not always best suited to capturing the complexity of systems-based approaches to public health issues (Barry et al., 2019), other scholars offer the counter-perspective that these are an appropriate trial design and can accommodate for the effects of local adaptation (Langford et al., 2015). Irrespective of the study design used, the findings of this study reinforce that the crux of evaluating whole-school interventions lies in the assessment of implementation, and the active use of implementation data in the interpretation of evaluation findings. While most studies (22 of 28) commented on implementation in some capacity, the majority focused on the *aggregate-level* of implementation among intervention schools. Only two studies (Andersen et al., 2019; Hamilton et al., 2005) provided a breakdown of implementation *between* intervention schools, finding that one-third or less of intervention schools classified as high-implementers, and up to one-half as low-implementers. These findings are consistent with previous literature, which demonstrates that multicomponent interventions, such as whole-school programmes, commonly encounter challenges with implementation (Durlak & DuPre, 2008). Both of these studies subsequently examined the impact of implementation on outcomes. For example, Andersen et al. (2019) identified that intervention schools classified as high-implementers demonstrated a significant 56% reduction in the odds of smoking when compared to the control; moderate-implementers a significant 30% reduction; and low-implementers no significant difference from the control condition. This association is mirrored in the literature, with a previous meta-analysis identifying that effect sizes were twice as high for intervention groups with high-levels of implementation as compared to those with low-levels of implementation (Durlak et al., 2011). Herein is emphasized the critical influence of implementation on outcomes. These findings evidence that an intervention evaluated as ineffective may be due to poor implementation of that intervention. This signifies that the intervention has deviated substantially from its intended design and this can lead to misleading conclusions about the *intended* intervention’s effectiveness (Barry et al., 2019). The findings of this study thus advocate for evaluations of whole-school interventions to not only assess implementation, as many of the included studies have done, but for the active use of this implementation data in the interpretation of evaluation findings. Given the ongoing challenges encountered in the implementation of whole-school interventions, the findings also highlight the need for training and support mechanisms to be incorporated into interventions to bolster implementation.

Consistent with previous literature, a preponderance of studies in this review have assessed implementation in relation to dosage and fidelity (Durlak, 2016). The remaining six components, however, remained largely neglected by studies. This meta-analysis illuminates one component in need of particular attention: the monitoring of the control condition. Only two studies monitored the implementation of preventive activities in the control condition (Dray et al., 2017; de Vries et al., 2006). These found that for several intervention strategies, there was no significant difference in the level of activities implemented by the intervention and control conditions, and that for certain intervention strategies, the control condition implemented *significantly more* activities than did the intervention condition. It is important to highlight that both of these studies described their control condition as “school-as-usual”. These findings emphasize the school as a complex and adaptive system in which a truly inactive control condition may be challenging to achieve. While a sensitivity analysis removing studies with an active control condition yielded findings that were unchanged from the overall meta-analysis results, the findings indicate that this study may have underestimated the number of studies that have described their control condition as “school-as-usual”, similar to the two studies referenced above, but have inadvertently involved active control conditions that would not have been accounted for in the sensitivity analysis. This means that evaluations of whole-school interventions may effectively be comparing two trial groups that are simply implementing *different* preventive activities, the level of which may remain unknown to trial authors and readers alike, unless explicitly measured. This study recommends that future evaluations monitor the preventive activities implemented by the control condition that may overlap with the whole-school intervention’s aim, as this would enable a more nuanced and accurate interpretation of evaluation findings. To facilitate this, this study advocates for greater investment in the development of standardized and comprehensive tools to measure implementation. One such tool that was recently developed by Vennegoor et al. (2023) to monitor the implementation of health-promoting schools’ activities is spotlighted. When used in both the intervention and control conditions, the tool can monitor all eight components of implementation and has preliminary evidence in support of its reliability and validity.

The findings of this study should be considered in light of several limitations. The first is the variability observed in the quality of included studies. Though most studies classified as either low or unclear risk for each of the risk-of-bias categories, the exception was Domain 4 (detection bias), where 25 of the 28 studies classified as high risk-of-bias. This was primarily due to the lack of information provided by studies on whether participants were blinded to the trial, in the broader context of the difficulty faced in blinding for this type of intervention. Secondly, while the protocol for this study outlined intentions to assess for publication bias using funnel plots, Egger’s test and trim-and-fill analyses, these could not be conducted due to an insufficient number of studies. Thirteen of the fourteen meta-analyses had less than ten studies, with best practice guidelines advising against these assessments in the case of less than 10 studies due to inadequate statistical power (Higgins et al., 2019). The third pertains to the high heterogeneity (>50%) observed for four of the outcomes meta-analyzed, creating challenges in meaningfully combining the effect estimates within these four outcome domains to draw definitive conclusions. The heterogeneity observed may be explained by various factors, including the inclusion of diverse samples, interventions that typify a whole-school approach in different ways, and the use of varying outcome measures by studies. It is worthwhile noting, however, that for the outcomes identified to be significantly different between the intervention and control conditions, two demonstrated low heterogeneity (cyber-bullying and cyber-aggression) and the other moderate heterogeneity (regular smoking). Traditional methods to further investigate heterogeneity, including subgroup analyses and meta-regression, could not be conducted due to the lack of studies for each outcome domain (Higgins et al., 2019). This similarly meant that this study was unable to address the variability in programme duration and explore its impact on intervention effectiveness. Finally, while previous reviews and the present study have focused on meta-analyzing individual-level outcomes, this study encourages future reviews to consider the inclusion of outcomes at broader levels of the socio-ecological model. For example, six studies reported on school-level outcomes, including school climate and safety (Bond et al., 2004a; Bonell et al., 2018; Cross et al., 2018; Shinde et al., 2018; Hunt et al., 2007; Sawyer et al., 2010a, 2010b). The inclusion of outcomes at the various levels of the socio-ecological model would be more aligned with the theoretical underpinnings of whole-school interventions, given that these interventions aspire for systems-level change.

## Conclusion

Whole-school interventions, informed by the Health-Promoting Schools Framework, offer a holistic, systems-based approach to promoting mental health and preventing risk behaviours among adolescents. Despite their potential in achieving these objectives, the evidence on their effectiveness is inconclusive, partly driven by the insufficient number of studies included in previous meta-analyses. This study performed an updated systematic review and meta-analysis on the effectiveness of whole-school interventions in promoting mental health and preventing risk behaviours in adolescents. The findings identified that whole-school interventions offer large population health benefits for three highly-prevalent issues among adolescents, reducing cyber-bullying, regular smoking and cyber-aggression by 25, 31 and 37%, respectively. However, no significant difference was found between trial groups for the remaining eleven outcomes, including mental disorders, positive mental health, alcohol and recreational drug use. These non-significant findings may be attributable to the suboptimal translation of the Health-Promoting Schools Framework into practice and the inadequate responsiveness of interventions to the local developmental needs of adolescents. While 22 of the 28 studies evaluated implementation in some capacity, a preponderance of studies assessed only implementation fidelity and dosage and only 2 studies examined the impact of implementation on outcomes. The ongoing challenges observed in the implementation of these complex interventions reinforces the need for high-quality training and support mechanisms to be embedded in interventions to bolster implementation. This study highlights that the crux of evaluating whole-school interventions lies not only in assessing the implementation of health-promoting activities in intervention and control conditions alike, but in the active use of implementation data to facilitate an accurate and nuanced interpretation of evaluation findings.

## Supplementary information

Supplementary Materials 1_Search Strategy

Supplementary Materials 2_RoB Assessments

Supplementary Materials 3_Individual Forrest Plots

Supplementary Materials 4_Baseline Outcomes

## Data Availability

The datasets generated and/or analyzed during the current study are not publicly available but are available from the corresponding author on reasonable request.
