# Supplementary material for: Systematic Review and Meta-analysis of the Effectiveness of Whole-school Interventions Promoting Mental Health and Preventing Risk Behaviours in Adolescence
Source: J Youth Adolesc. 2025 Jan 27;54(2):271–89. doi: 10.1007/s10964-025-02135-6 (PMC11807013; doi:10.1007/s10964-025-02135-6)
Supplement: Supplementary file 1 — Supplementary Materials 1_Search Strategy [file 10964_2025_2135_MOESM1_ESM.docx]

**Systematic Review and Meta-Analysis of the Effectiveness of Whole-School Interventions Promoting Mental Health and Preventing Risk Behaviours in Adolescence**

SUPPLEMENTARY MATIERIALS 1:
MEDLINE SEARCH STRATEGY

**Ovid MEDLINE(R)**1946 to July 31, 2023

| **#** | **Searches** | **Results** |
| --- | --- | --- |
| 1 | curriculum/ or schools/ or School Teachers/ or School Health Services/ | 152778 |
| 2 | (curriculum or curricula* or ((classroom or class room or schoolbased or school based or school setting* or whole school or school approach or school policies or school policy or school health polic* or school wide or schoolwide or school delivered or school led or teacher led or school teacher* or school climate or school community or teaching staff* or school staff* or core subject?) adj8 (intervention* or program*))).mp. | 119884 |
| 3 | ((universal or school or teacher-led or teacherled or system wide or systemwide or population wide or populationwide or student wide or studentwide or population based or populationbased or student based or studentbased) adj3 (intervention* or program*)).mp. | 19116 |
| 4 | ((embed* or delivered or implemented or integrated or incorporated) adj4 (school? or teacher* or highschool*)).mp. | 3576 |
| 5 | 1 or 2 or 3 or 4 | 188382 |
| 6 | Adolescent/ or Students/ | 2263429 |
| 7 | (adolescen* or teen* or secondary school* or school student? or school girl? or school boy? or high school or middle school or grade school).mp. | 2277508 |
| 8 | ((child* or boy? or girl?) adj aged adj3 (12 years or 13 years or 14 years or 15 years or 16 years or 17 years or 18 years or twelve years or thirteen years or fourteen years or fifteen years or sixteen years or seventeen years or eighteen years)).mp. | 26602 |
| 9 | ((child* or boy? or girl?) adj4 (12 year? Old? or 13 year? Old? or 14 year? Old? or 15 year? Old? or 16 year? Old? or 17 year? Old? or 18 year? Old? or 12 years of age or 13 years of age or 14 years of age or 15 years of age or 16 years of age or 17 years of age or 18 years of age)).mp. | 55782 |
| 10 | ((child* or boy? or girl?) adj3 (age* twelve or age* thirteen or age* fourteen or age* fifteen or age* sixteen or age* seventeen or age* eighteen or age* 12 or age* 13 or age* 14 or age* 15 or age* 16 or age* 17 or age* 18 or age* of twelve or age* of thirteen or age* of fourteen or age* of fifteen or age* of sixteen or age* of seventeen or age* of eighteen or age* of 12 or age* of 13 or age* of 14 or age* of 15 or age* of 16 or age* of 17 or age* of 18)).mp. | 43944 |
| 11 | 6 or 7 or 8 or 9 or 10 | 2348221 |
| 12 | Mental health/ or Emotions/ or Psychological wellbeing/ or Personal satisfaction/ or mindfulness/ or resilience, psychological/ or happiness/ or hope/ or Emotional regulation/ or affect/ or interpersonal relations/ or quality of life/ or Social integration/ or Social cohesion/ or Social adjustment/ or Social responsibility/ or Happiness/ or Social skills/ or Social identification/ or Self-concept/ or Self-efficacy/ or Sense of coherence/ | 636429 |
| 13 | (mental health or psychological health or stress or emotion* or personal satisfaction or quality of life or interpersonal relation* or social integration or social cohesion or social adjustment or social responsibility or happiness or social skill* or social identification or self-concept or self-efficacy or sense of coherence or social wellbeing or social well-being or life satisfaction or self-esteem).mp. | 1896384 |
| 14 | ((wellness or wellbeing or well-being or empowerment or resilience or resilient or flourishing) adj5 (psychological* or mental* or emotional*)).mp. | 41922 |
| 15 | mental disorders/ or anxiety disorders/ or obsessive-compulsive disorder/ or panic disorder/ or phobic disorders/ or phobia, social/ or "disruptive, impulse control, and conduct disorders"/ or dissociative disorders/ or dissociative identity disorder/ or "feeding and eating disorders"/ or anorexia nervosa/ or avoidant restrictive food intake disorder/ or binge-eating disorder/ or bulimia nervosa/ or diabulimia/ or "feeding and eating disorders of childhood"/ or mood disorders/ or "bipolar and related disorders"/ or depressive disorder/ or cyclothymic disorder/ or attention deficit disorder with hyperactivity/ or conduct disorder/ or child behavior disorders/ or reactive attachment disorder/ or schizophrenia, childhood/ or personality disorders/ or antisocial personality disorder/ or borderline personality disorder/ or compulsive personality disorder/ or dependent personality disorder/ or histrionic personality disorder/ or paranoid personality disorder/ or passive-aggressive personality disorder/ or schizoid personality disorder/ or schizotypal personality disorder/ or "schizophrenia spectrum and other psychotic disorders"/ or affective disorders, psychotic/ or psychotic disorders/ or psychoses, substance-induced/ or schizophrenia/ or schizophrenia, catatonic/ or schizophrenia, disorganized/ or schizophrenia, paranoid/ or schizophrenia, treatment-resistant/ or somatoform disorders/ or body dysmorphic disorders/ or body integrity identity disorder/ or conversion disorder/ or factitious disorders/ or munchausen syndrome/ or munchausen syndrome by proxy/ or substance-related disorders/ or alcohol-related disorders/ or alcoholic intoxication/ or alcoholism/ or binge drinking/ or psychoses, alcoholic/ or amphetamine-related disorders/ or cocaine-related disorders/ or inhalant abuse/ or marijuana abuse/ or "marijuana use"/ or narcotic-related disorders/ or neonatal abstinence syndrome/ or phencyclidine abuse/ or substance abuse, intravenous/ or substance abuse, oral/ or "trauma and stressor related disorders"/ or adjustment disorders/ or stress disorders, traumatic/ or stress disorders, post-traumatic/ or stress, psychological/ or burnout, psychological/ or adolescent behavior/ or underage drinking/ or behavioral symptoms/ or affective symptoms/ or delusions/ or depersonalization/ or depression/ or obsessive behavior/ or paranoid behavior/ or problem behavior/ or self-injurious behavior/ or self mutilation/ or suicide/ or suicidal ideation/ or suicide, attempted/ or suicide, completed/ or impulsive behavior/ or compulsive behavior/ or "marijuana use"/ or marijuana smoking/ or "recreational drug use"/ or risk-taking/ or bullying/ or cyberbullying/ | 1134951 |
| 16 | ((mental or anxiety or neurotic or obsessive-compulsive or compulsive-obsessional or panic or phobic or bipolar or disruptive or impulse control or conduct or dissociative or identity or eating or mood or depressive or dysthymic or affective or attention deficit or behavio* or hyperactivity or autism spectrum or autistic or paraphilic or personality or psychotic or paranoid or somatoform or somati#ation or somatic symptom or psychophysiologic* or psychosomatic or body dysmorphic or body integrity identity or stressor related or adjustment or traumatic stress or post-traumatic or posttraumatic or acute traumatic or emotional or disruptive mood dysregulation or substance induced or medication induced or alcohol induced or substance related or alcohol related or drug induced or hoarding or attachment or conversion or bodily distress or functional movement or functional neurological or functional neurologic symptom or sleep wake or sleep initiation or sleep maintenance or insomnia or oppositional defiant or intermittent explosive or factitious or psychoneurotic or manic or cyclothymic or autophag* or schizoaffective or unipolar or delusional or addiction or abnormal psychology) adj disorder*).mp. | 797872 |
| 17 | 12 or 13 or 14 or 15 or 16 | 2768293 |
| 18 | (randomized controlled trial or controlled clinical trial).pt. | 687637 |
| 19 | (randomi?ed or placebo or randomly).ab. or trial.ti. | 1090853 |
| 20 | clinical trials as topic.sh. | 201126 |
| 21 | 18 or 19 or 20 | 1420530 |
| **22** | **5 and 11 and 17 and 21** | **2945** |
| **23** | **limit 22 to english language** | **2890** |
| **24** | **limit 22 to (case reports or comment or editorial or letter or news or newspaper article)** | **17** |
| **25** | **22 not 24** | **2928** |
| **26** | **limit 22 to ("review" or "systematic review")** | **160** |
| **27** | **22 not 26** | **2785** |
