# Supplementary material for: Systematic Review and Meta-analysis of the Effectiveness of Whole-school Interventions Promoting Mental Health and Preventing Risk Behaviours in Adolescence
Source: J Youth Adolesc. 2025 Jan 27;54(2):271–89. doi: 10.1007/s10964-025-02135-6 (PMC11807013; doi:10.1007/s10964-025-02135-6)
Supplement: Supplementary file 2 — Supplementary Materials 2_RoB Assessments [file 10964_2025_2135_MOESM2_ESM.docx]

**Systematic Review and Meta-Analysis of the Effectiveness of Whole-School Interventions Promoting Mental Health and Preventing Risk Behaviours in Adolescence**

SUPPLEMENTARY MATERIAL 2:
Risk-of-Bias Assessments

Contents

[Overall approach to risk-of-bias assessments 2](#_Toc175922036)

[Individual risk-of-bias assessments 2](#_Toc175922037)

[Allara et al. 2019 2](#_Toc175922038)

[Andersen et al. 2015 3](#_Toc175922039)

[Bond et al. 2004 4](#_Toc175922040)

[Bonell et al. 2018 4](#_Toc175922041)

[Bonnesen et al. 2023 4](#_Toc175922042)

[Cross et al. 2018 5](#_Toc175922043)

[Shinde et al. 2018 6](#_Toc175922044)

[Perry et al. 2009 7](#_Toc175922045)

[Dray et al. 2017 8](#_Toc175922046)

[Sawyer et al. 2010 8](#_Toc175922047)

[Gorini et al. 2014 9](#_Toc175922048)

[Karna et al. 2012 10](#_Toc175922049)

[De Vries et al. 2006 (Denmark) 10](#_Toc175922050)

[Perry et al. 2003 11](#_Toc175922051)

[Cross et al. 2015 11](#_Toc175922052)

[De Vries et al. 2006 (Finland) 12](#_Toc175922053)

[Hunt et al. 2007 12](#_Toc175922054)

[Foshee et al. 1998 13](#_Toc175922055)

[Johnson et al. 2017 13](#_Toc175922056)

[Schofield et al. 2003 13](#_Toc175922057)

[Wen et al. 2010 14](#_Toc175922058)

[Larsen et al. 2023 15](#_Toc175922059)

[Stevens et al. 2000 15](#_Toc175922060)

[Rahman et al. 1998 16](#_Toc175922061)

[Skarstrand et al. 2013 16](#_Toc175922062)

[Wolfe et al. 2009 17](#_Toc175922063)

# Overall approach to risk-of-bias assessments

| Category | Considerations |
| --- | --- |
| Selection bias  (Section 1a, 1b) | For section 1a (random sequence generation), if a study specified a method of generating a truly random sequence it was classified as low risk; if a study described the trial was randomised but did not specify what method was used to generate the random sequence it was classified as unclear risk, and if a study used a predictable sequence or demonstrated marked baseline differences between the intervention and control groups (beyond likelihood of chance) it was classified as high risk. For section 1b (allocation concealment), if a study specified that *all* clusters had been randomised at the start of the study and did not demonstrate marked differences between the intervention and control groups, it was classified as low risk. |
| Performance bias  (Section 2) | If trial groups were blinded and an appropriate analysis strategy used, a study was classified as low risk. If a study did not specifically reference the presence or absence of deviations, it was classified as unclear risk. If a study described deviations to the protocol, we considered whether these deviations were balanced between the intervention and control groups, the likely impact of the deviation on the study results, and whether the study has used an appropriate analysis strategy, in determining the risk posed. |
| Attrition bias  (Section 3) | If a study analysed data from all clusters included at the start of the trial, and all or nearly all participants were analysed within each cluster, a study was classified as low risk. If a study (i) did not analyse data from all clusters, (ii) there was marked loss-to-follow-up within each cluster, (iii) or there was differential loss-to-follow-up between groups, we considered whether the study used an appropriate analysis strategy in determining the risk posed. |
| Detection bias  (Section 4) | Given the intervention type, it is challenging to blind participants to the trial. Hence, if a study did not explicitly describe that participants and outcome assessors (often participants themselves via self-report measures) were blinded to the trial, it would be classified as high risk. If a study did explicitly describe that participants and outcome assessors were blinded to the trial, we considered how effective blinding was in determining the risk posed. |
| Reporting bias  (Section 5) | If a study referenced a protocol and reported results consistent with this protocol, it was classified as low risk. If a study did not reference a protocol, it was classified as unclear risk. If a study referenced a protocol but the results presented were discordant with this protocol, or if there was evidence that results were derived from multiple outcome measures or analyses, it was classified as high risk. |

# Individual risk-of-bias assessments

## Allara et al. 2019

| Randomisation Process (Selection Bias) | Low | Schools were stratified to form strata of schools presenting with similar characteristics. Within each stratum, schools were randomly assigned to the experimental or control arm using a computer-generated list of random numbers. |
| --- | --- | --- |
| Timing of identification or recruitment of participants (Selection Bias) | Low | Allocation procedure was carried out centrally by independent contractors at the start of the study. |
| Deviations from intended interventions  (Performance Bias) | High | Per-protocol analysis: “Just after the randomization procedure and before the start of the program, three control schools implementing other prevention activities were switched to the intervention group. Analyses excluded these participants who did not receive their assigned intervention”. |
| Missing Outcome Data  (Attrition Bias) | High | “Just after the randomization procedure and before the start of the program, three control schools implementing other prevention activities were switched to the intervention group.” These schools (n = 3) and their matched counterparts (n = 3) were excluded from the analyses. States that multiple imputation was used, but that it did not account for these excluded clusters. |
| Measurement of the Outcome (Detection Bias) | High | Not indicated if outcome assessors (participants) aware they are in a trial and not feasible to blind outcome assessors (participants) to intervention. |
| Selection of the Reported Result (Reporting Bias) | Some concerns | Protocol registered retrospectively, after data collection had begun. |

## Andersen et al. 2015

| Randomisation Process  (Selection Bias) | Low | "Stratified simple randomization procedure by municipality. Within municipalities, each school had an equal probability of being either a control or an intervention school. Randomization was conducted by drawing lots”. |
| --- | --- | --- |
| Timing of identification or recruitment of participants (Selection Bias) | Low | Allocation of schools to intervention or control condition occurred at the start of the study. |
| Deviations from intended interventions  (Performance Bias) | Some concerns | Not indicated if participants aware they were in a trial. Not feasible to blind to this intervention.  Deviations not discussed. Intention-to-treat analysis performed. |
| Missing Outcome Data  (Attrition Bias) | High | "Three schools withdrew after randomization, leaving: 51 schools in the intervention group, with an average number of 49.6 students per school; and 43 schools in the control group, with an average number of 45.0 students per school.” Withdrawn schools not included in analysis. |
| Measurement of the Outcome (Detection Bias) | High | Not indicated if participants (outcome assessors) aware of the trial and not feasible to blind outcome assessors (participants) to intervention. |
| Selection of the Reported Result (Reporting Bias) | Low | Design paper lists same primary outcome. |

## Bond et al. 2004

| Randomisation Process  (Selection Bias) | High | "Using simple random sampling, 12 schools were selected from the ‘‘intervention’’ districts and 12 from the ‘‘control’’ districts. Intervention and control schools thus selected from different pre-specified geographical districts. Randomisation method also not detailed. |
| --- | --- | --- |
| Timing of identification or recruitment of participants (Selection Bias) | Low | Schools allocated to intervention and control conditions at the beginning of the study. |
| Deviations from intended interventions  (Performance Bias) | Some concerns | Does not indicate if participants aware they are in a trial. Not feasible to blind participants to intervention. Doesn’t specify if there were deviations. Intention-to-treat analysis used. |
| Missing Outcome Data  (Attrition Bias) | Low | Attrition across the waves was less than 3%, 8%, and 10% respectively with no differential response rate between intervention and control groups at the subsequent waves (98% v 96%; 92% v 92%, and 90% v 89% respectively). |
| Measurement of the Outcome (Detection Bias) | High | Not indicated if participants (outcome assessors) aware of the trial and not feasible to blind outcome assessors (participants) to intervention. |
| Selection of the Reported Result (Reporting Bias) | High | No protocol available. Multiple analyses reported for certain outcomes. |

## Bonell et al. 2018

| Randomisation Process  (Selection Bias) | Low | Stratified randomisation. Sequence allocation generated using Stata's ralloc command. |
| --- | --- | --- |
| Timing of identification or recruitment of participants (Selection Bias) | Some concerns | “We randomly allocated schools to the intervention group or the control group immediately after baseline surveys”. |
| Deviations from intended interventions  (Performance Bias) | Some concerns | Not indicated whether participants aware they were in a trial. Difficult to blind to intervention in this case. Does not detail if there were deviations from the intended intervention. Intention-to-treat analysis used. |
| Missing Outcome Data  (Attrition Bias) | Low | No loss of clusters and no difference in loss to follow-up between trial groups. |
| Measurement of the Outcome (Detection Bias) | High | Not indicated if participants (outcome assessors) aware of the trial and not feasible to blind outcome assessors (participants) to intervention. |
| Selection of the Reported Result (Reporting Bias) | Low | Study protocol details outcome measures reported on in this paper. |

## Bonnesen et al. 2023

| Randomisation Process  (Selection Bias) | Low | "Randomisation conducted using a computer-based random number generator.” |
| --- | --- | --- |
| Timing of identification or recruitment of participants (Selection Bias) | Low | Randomisation of schools to intervention and control conditions occurred at the start of the study. |
| Deviations from intended interventions  (Performance Bias) | Some concerns | Does not indicate if participants aware they were in a trial. Not feasible to blind participants to intervention. Does not specify any deviations from the protocol. Intention-to-treat analysis. |
| Missing Outcome Data  (Attrition Bias) | High | Complete-case analyses had 3 schools less than baseline, and analyses with multiple imputation had 2 schools less than baseline. One-third of students were lost-to-follow-up as they no longer attended one of the participating schools at the time of the follow-up or because their high schools were unable to find time to conduct the survey among students. |
| Measurement of the Outcome (Detection Bias) | High | Not indicated if participants (outcome assessors) aware of the trial and not feasible to blind outcome assessors (participants) to intervention. |
| Selection of the Reported Result (Reporting Bias) | Some concerns | "The primary outcome of the trial (well-being) as well as the outcomes of the four other pathways to well-being will be reported elsewhere." These reports could not be located, acknowledging the possibility that they are yet to be published. |

## Cross et al. 2018

| Randomisation Process  (Selection Bias) | Some concerns | Does not specify method of generating random sequence. |
| --- | --- | --- |
| Timing of identification or recruitment of participants (Selection Bias) | Some concerns | One additional school was added as a comparison school when the study cohort was in Grade 8 to offset a school that was originally included as a comparison school but requested resources and staff professional development. This school’s data are not included in these analyses. Thus, one additional school had been added as a comparison school after the initial randomisation process. |
| Deviations from intended interventions  (Performance Bias) | Some concerns | Not feasible to blind participants to intervention. Does not specifically mention deviations. Appears to be modified ITT, excluding participants with missing outcome data. |
| Missing Outcome Data  (Attrition Bias) | Low | Reasonable attrition rates and comparable between groups. |
| Measurement of the Outcome (Detection Bias) | High | Not indicated if participants (outcome assessors) aware of the trial and not feasible to blind outcome assessors (participants) to intervention. |
| Selection of the Reported Result (Reporting Bias) | Some concerns | No protocol available. |

##

## Shinde et al. 2018

| Randomisation Process  (Selection Bias) | Low | The schools were randomly assigned to the three interventions in a 1:1:1 ratio using minimisation, stratified by type of school, school size, and gender composition. The random allocation was done by an independent statistician. |
| --- | --- | --- |
| Timing of identification or recruitment of participants (Selection Bias) | Low | The schools were randomly assigned to the three intervention conditions at the beginning of the study. |
| Deviations from intended interventions  (Performance Bias) | Low | "All the authors, apart from the trial statistician and data manager, were masked  until the trial groups were unblinded in the presence of both the Trial Steering and Data Safety and Monitoring Committees on Oct 17, 2016. Intention-to-treat analysis was used." |
| Missing Outcome Data  (Attrition Bias) | Low | All schools recruited participated in baseline and follow-up surveys. 60% completion rate of baseline surveys and 60-70% of endpoint surveys. Analyses by intention to treat.  Sensitivity analyses restricted to participants who completed all three surveys showed similar results for primary, secondary, and exploratory outcomes |
| Measurement of the Outcome (Detection Bias) | Some concerns | "The trial groups were unblinded in a joint meeting of the Trial Steering and Data Safety and Monitoring Committees on October 26, 2017" (this is post-intervention). However, does not elaborate on this so it is difficult to ascertain how successful the blinding process was. |
| Selection of the Reported Result (Reporting Bias) | Low | Outcomes measured and presented align with those described in the study protocol. |

## Malmberg et al. 2014

| Randomisation Process  (Selection Bias) | Low | An independent statistician performed the allocation before baseline assessment and randomly assigned the schools to intervention or control conditions. Randomization was carried out using a blocked randomization scheme (block size 6), and was stratified by the level of education that the schools offered. |
| --- | --- | --- |
| Timing of identification or recruitment of participants (Selection Bias) | Low | Schools allocated to intervention and control conditions at the beginning of the study. |
| Deviations from intended interventions  (Performance Bias) | Some concerns | Not clearly defined if participants were aware they were in a trial. Not feasible to blind participants given nature of the intervention.  Not specifically mentioned if deviations from the protocol occurred. Intention-to-treat analysis with multiple imputation. |
| Missing Outcome Data  (Attrition Bias) | High | "Just before the 32-month follow-up (T3), one school from the control condition and one school from the e-learning condition dropped out of  the study due to practical considerations. As adolescents were included by school participation, all students of these schools were lost to follow-up. A total of 2340 adolescents  of the remaining 21 schools participated at T3  (response rate out of n = 3542 = 66.1%). Adolescents lost to follow-up were less likely to be in pre-university education and more likely to be in higher general education or a combination of pre-university education and higher general education [odds ratio (OR) = 1.13, 95% confidence interval (CI) = 1.07, 1.20, P < 0.0001] compared to adolescents who completed the 32-month follow-up assessment”. |
| Measurement of the Outcome (Detection Bias) | High | Not indicated if outcome assessors (participants) aware they are in a trial and not feasible to blind outcome assessors (participants) to intervention. |
| Selection of the Reported Result (Reporting Bias) | High | "We have not reported the statistics of the other two time-points and the other outcomes that have been measured and included in our trial registration in the present paper." These also could not be found in future publications. |

## Perry et al. 2009

| Randomisation Process  (Selection Bias) | Some concerns | No information provided on how random allocation sequence was generated. |
| --- | --- | --- |
| Timing of identification or recruitment of participants (Selection Bias) | Low | Schools allocated to intervention and control conditions at the beginning of the study. |
| Deviations from intended interventions  (Performance Bias) | Some concerns | Not specifically referenced whether participants aware they are in a trial. Not feasible to blind participants to intervention. Not specifically referenced if deviations from the intended intervention. Does not specify if intention-to-treat analysis was used. No information on whether intention-to-treat analysis was used. |
| Missing Outcome Data  (Attrition Bias) | High | Two schools (one intervention and one control) did not participate in follow-up surveys due to conflicting schedules. Three schools (one intervention and two control) also did not allow grade 10 students to participate due to exams. High levels of attrition, with missing data higher among students who reported using tobacco. |
| Measurement of the Outcome (Detection Bias) | High | Not indicated if outcome assessors (participants) aware they are in a trial and not feasible to blind outcome assessors (participants) to intervention. |
| Selection of the Reported Result (Reporting Bias) | Some concerns | No protocol available |

## Dray et al. 2017

| Randomisation Process  (Selection Bias) | Low | "Schools were randomised in Microsoft Excel using a random number function in a 20:12 block design ratio (20 intervention; 12 control)”. |
| --- | --- | --- |
| Timing of identification or recruitment of participants (Selection Bias) | Low | Schools were allocated to intervention and control conditions at the beginning of the study. |
| Deviations from intended interventions  (Performance Bias) | Some concerns | "Additionally, informed student consent  for participation in the evaluation component of the study, will be required from each participant prior to completion of student surveys at each point of data collection. Schools, parents of students and enrolled students will not be blinded to study group allocation.” Does not explicitly reference any deviations from study protocol. Presented main results as complete-case analyses (without multiple imputation) alongside ITT with multiple imputation. |
| Missing Outcome Data  (Attrition Bias) | Low | Students from all intervention and control schools provided outcome data. About 30% of the baseline participants were lost-to-follow-up.  Study provided ITT with multiple imputation as a sensitivity analysis. |
| Measurement of the Outcome (Detection Bias) | High | Not indicated if outcome assessors (participants) aware they are in a trial and not feasible to blind outcome assessors (participants) to intervention. |
| Selection of the Reported Result (Reporting Bias) | Low | Outcomes prespecified in protocol. |

## Sawyer et al. 2010

| Randomisation Process  (Selection Bias) | Some concerns | No details on how random allocation sequence was generated. |
| --- | --- | --- |
| Timing of identification or recruitment of participants (Selection Bias) | Low | Schools were allocated by a research assistant who was blind to the groups to which schools were being allocated. |
| Deviations from intended interventions  (Performance Bias) | Some concerns | Informed consent was obtained from all participants and their parents prior to the baseline assessment. Not feasible to blind to this type of intervention. Deviations from the protocol were not specifically referenced. Intention-to-treat analysis used. |
| Missing Outcome Data  (Attrition Bias) | High | By the second-year follow-up, high levels of attrition with attrition greater in students who reported higher depressive scores at baseline. |
| Measurement of the Outcome (Detection Bias) | High | Not indicated if outcome assessors (participants) aware they are in a trial and not feasible to blind outcome assessors (participants) to intervention. |
| Selection of the Reported Result (Reporting Bias) | Some concerns | Does not reference protocol. |

## Gorini et al. 2014

| Randomisation Process  (Selection Bias) | Low | "Schools were centrally randomized to the experimental or control arm using a random number generator.” |
| --- | --- | --- |
| Timing of identification or recruitment of participants (Selection Bias) | Some concerns | "Schools allocated to intervention and control conditions at the beginning of the study. After randomisation, three schools allocated in the control group refused to comply with their assignment to the control condition so were included in the intervention condition, but these schools and their paired comparison schools were excluded from the main analyses.” |
| Deviations from intended interventions  (Performance Bias) | High | Does not specify if participants aware they are in a trial. Not feasible to blind to intervention.  References that 7 schools violated the protocol post-randomisation and thus were excluded from analyses. Modified ITT analysis used. |
| Missing Outcome Data  (Attrition Bias) | Some concerns | "7 clusters were removed from analyses post-randomisation as they violated the study protocol. Of the clusters included (n=13), lost-to-follow-up was 20-25%. We conducted a comparison of the baseline characteristics  between the samples with and without the 7 non-compliant schools. There were no differences between these two samples, except  for the distribution of gender (fewer girls in the intervention group in the sample without the 7 schools). Moreover, in an additional (sensitivity) analysis (data not shown) main results did not differ significantly including the 7 non-compliant schools.” Documented reasons for not including the non-compliant schools (protocol violations), but no documented reasons for why individual participants within clusters did not provide outcome data and the study did not correct for loss-to-follow-up with multiple imputation. |
| Measurement of the Outcome (Detection Bias) | High | Not indicated if outcome assessors (participants) aware they are in a trial and not feasible to blind outcome assessors (participants) to intervention. |
| Selection of the Reported Result (Reporting Bias) | Low | Outcome measures detailed in protocol. No outcomes described in the protocol that weren’t reported, but several extra outcomes included in the paper that weren't detailed in the protocol. |

## Karna et al. 2012

| Randomisation Process (Selection Bias) | Some concerns | No information on how random allocation sequence was generated. |
| --- | --- | --- |
| Timing of identification or recruitment of participants (Selection Bias) | Low | Schools allocated to intervention and control conditions at the beginning of the study. |
| Deviations from intended interventions  (Performance Bias) | Some concerns | Does not specifically state if participants aware of trial. Not feasible to blind participants to intervention status. Does not specify if deviations from protocol occurred. Does not specify if intention-to-treat analysis was used. Insufficient detail provided on analysis method to discern if substantial impact. |
| Missing Outcome Data  (Attrition Bias) | High | Seven control and two intervention schools dropped out without providing data and one school participated only in baseline data collection. This data subsequently excluded from analysis. Students who dropped out had poorer bullying outcomes vs those who completed trial. |
| Measurement of the Outcome (Detection Bias) | High | Not indicated if outcome assessors (participants) aware they are in a trial and not feasible to blind outcome assessors (participants) to intervention. |
| Selection of the Reported Result (Reporting Bias) | Some concerns | No available protocol for trial. |

## De Vries et al. 2006 (Denmark)

| Randomisation Process  (Selection Bias) | Some concerns | No information on how random allocation sequence was generated. |
| --- | --- | --- |
| Timing of identification or recruitment of participants (Selection Bias) | Low | Schools were allocated to intervention and control conditions at the beginning of the study. |
| Deviations from intended interventions  (Performance Bias) | Some concerns | Does not indicate if participants aware of trial status. Not feasible to blind participants to intervention. Listed deviations of the intervention by participating country, though these are unlikely to have occurred due to the trial context. Does not specify if intention-to-treat analysis was used. |
| Missing Outcome Data  (Attrition Bias) | High | At final follow-up, Denmark reported a reversed pattern of response rates between the experimental and control groups (41 versus 60%). Multiple imputation and sensitivity analyses not provided. |
| Measurement of the Outcome (Detection Bias) | High | Not indicated if outcome assessors (participants) aware they are in a trial and not feasible to blind outcome assessors (participants) to intervention. |
| Selection of the Reported Result (Reporting Bias) | Some concerns | No protocol available for the study. |

## Perry et al. 2003

| Randomisation Process  (Selection Bias) | Some concerns | Does not specify method of generating random sequence. |
| --- | --- | --- |
| Timing of identification or recruitment of participants (Selection Bias) | Low | Schools allocated to intervention and control conditions at the beginning of the study. |
| Deviations from intended interventions  (Performance Bias) | Some concerns | Does not specify if participants aware they are in a trial. Not feasible to blind participants to intervention status. Does not specify deviations from the intended protocol. Does not specifically state if intention-to-treat analysis was used. |
| Missing Outcome Data  (Attrition Bias) | High | Approximately 65% of students completed all three assessments. No differential attrition between trial groups for primary outcomes; however, those who did not complete all three assessments had higher levels of violent behaviour and drug use at baseline assessment. |
| Measurement of the Outcome (Detection Bias) | High | Not indicated if participants (outcome assessors) aware of the trial and not feasible to blind outcome assessors (participants) to intervention. |
| Selection of the Reported Result (Reporting Bias) | Some concerns | No protocol available. |

## Cross et al. 2016

| Randomisation Process  (Selection Bias) | Some concerns | No details provided on how the random sequence allocation was generated. |
| --- | --- | --- |
| Timing of identification or recruitment of participants (Selection Bias) | Low | Schools allocated to intervention and control conditions at the beginning of the study. |
| Deviations from intended interventions  (Performance Bias) | Some concerns | Does not specify if participants aware of trial status. Not feasible to blind participants to intervention status. Does not specify if there were deviations from the intended protocol.  Does not specify whether ITT analysis used. |
| Missing Outcome Data  (Attrition Bias) | Low | All clusters provided outcome data at each follow-up. Attrition 85% in both trial groups. |
| Measurement of the Outcome (Detection Bias) | High | Not indicated if outcome assessors (participants) aware they are in a trial and not feasible to blind outcome assessors (participants) to intervention. |
| Selection of the Reported Result (Reporting Bias) | Some concerns | No protocol available. |

##

## De Vries et al. 2006 (Finland)

| Randomisation Process  (Selection Bias) | Some concerns | Does not provide information on how random allocation sequence was generated. |
| --- | --- | --- |
| Timing of identification or recruitment of participants (Selection Bias) | Low | Schools allocated to intervention and control conditions at the start of the study. |
| Deviations from intended interventions  (Performance Bias) | Some concerns | Does not specify if participants aware they are in a trial. Not feasible to blind participants to intervention. Deviations to intervention unlikely due to trial context. Does not specifically state if intention-to-treat analysis was used. |
| Missing Outcome Data  (Attrition Bias) | High | Two control schools dropped out by final follow-up, resulting in attrition of 27% in intervention group compared with 46% in the control group. |
| Measurement of the Outcome (Detection Bias) | High | Not indicated if outcome assessors (participants) aware they are in a trial and not feasible to blind outcome assessors (participants) to intervention. |
| Selection of the Reported Result (Reporting Bias) | Some concerns | Does not reference a study protocol. |

## Hunt et al. 2007

| Randomisation Process  (Selection Bias) | Some concerns | Does not detail how the random allocation sequence was generated. |
| --- | --- | --- |
| Timing of identification or recruitment of participants (Selection Bias) | Some concerns | Two schools in the intervention condition dropped out of the study in the early stages of the project (due to time constraints). One additional intervention school was recruited in 2002 to balance the study design. |
| Deviations from intended interventions  (Performance Bias) | Some concerns | No indication if participants aware they are in a trial. Not feasible to blind participants to intervention status. Does not detail deviations from the intended protocol. Does not indicate analysis method, if it is ITT or otherwise. |
| Missing Outcome Data  (Attrition Bias) | High | Two intervention schools dropped out of the study. 444 participants responded to baseline survey, 400 at one-year follow-up. Does not indicate that multiple imputation performed or that study authors undertook sensitivity analysis. |
| Measurement of the Outcome (Detection Bias) | High | Not indicated if outcome assessors (participants) aware they are in a trial and not feasible to blind outcome assessors (participants) to intervention. |
| Selection of the Reported Result (Reporting Bias) | Some concerns | Does not indicate protocol or design paper. |

## Foshee et al. 1998

| Randomisation Process  (Selection Bias) | Some concerns | No details on how random allocation sequence was generated. |
| --- | --- | --- |
| Timing of identification or recruitment of participants (Selection Bias) | Low | Schools were allocated to intervention and control conditions at the beginning of the study. |
| Deviations from intended interventions  (Performance Bias) | Some concerns | Does not detail if participants aware they are in a trial. Not feasible to blind participants to intervention. References change to school health services component as adolescents’ low number of adolescents accessing support groups at school, though this is unlikely due to the trial context. Does not indicate if intention-to-treat analysis was used. |
| Missing Outcome Data  (Attrition Bias) | Low | References that no schools dropped out of the intervention. 90% of baseline sample completed follow-up data collection. |
| Measurement of the Outcome (Detection Bias) | High | Not indicated if outcome assessors (participants) aware they are in a trial and not feasible to blind outcome assessors (participants) to intervention. |
| Selection of the Reported Result (Reporting Bias) | Some concerns | No protocol available. |

## Johnson et al. 2017

| Randomisation Process  (Selection Bias) | Low | "Randomised performed using the randomisation function in Excel." |
| --- | --- | --- |
| Timing of identification or recruitment of participants (Selection Bias) | Low | Individual classes allocated to intervention and control conditions at the beginning of the study. |
| Deviations from intended interventions  (Performance Bias) | Some concerns | Does not indicate if participants aware of trial status. Participants could not be blinded to the allocated treatment group. Does not indicate if deviations from the intended protocol. Does not indicate if intention-to-treat analysis was used. |
| Missing Outcome Data  (Attrition Bias) | Low | Does not specify if all clusters contributed outcome data. Lost-to-follow-up ~20-25%.  Compensated via analysis: inclusion of cases with missing data via maximum likelihood estimation, with baseline measures entered as covariates. |
| Measurement of the Outcome (Detection Bias) | High | Not indicated if outcome assessors (participants) aware they are in a trial and not feasible to blind outcome assessors (participants) to intervention. |
| Selection of the Reported Result (Reporting Bias) | Some concerns | No protocol available for study. |

## Schofield et al. 2003

| Randomisation Process  (Selection Bias) | Some concerns | No information provided on how random sequence allocation was generated. |
| --- | --- | --- |
| Timing of identification or recruitment of participants (Selection Bias) | Low | Schools were allocated to intervention and control conditions at the beginning of the study. |
| Deviations from intended interventions  (Performance Bias) | Some concerns | Does not indicate if participants aware of trial status. Difficult to blind participants to this type of intervention. Does not detail deviations from intended protocol. Does not define if intention-to-treat analysis was used. |
| Missing Outcome Data  (Attrition Bias) | High | Two control schools dropped out post-randomisation. High rates of attrition. Students who dropped out were more likely to smoke at baseline. Does not mention multiple imputation of missing results or sensitivity analyses. |
| Measurement of the Outcome (Detection Bias) | High | Not indicated if outcome assessors (participants) aware they are in a trial and not feasible to blind outcome assessors (participants) to intervention. |
| Selection of the Reported Result (Reporting Bias) | High | Discordant reporting of outcomes when compared to trial protocol. |

## Wen et al. 2010

| Randomisation Process  (Selection Bias) | Low | The randomisation was performed using a random number generation method by a statistician who was uninvolved in this study. |
| --- | --- | --- |
| Timing of identification or recruitment of participants (Selection Bias) | Low | The randomisation was performed by a statistician who was uninvolved in this study and also blinded to schools. |
| Deviations from intended interventions  (Performance Bias) | Low | "Because of the nature of this study, only students, neither research assistants nor school administrators, were blind to intervention allocation.” |
| Missing Outcome Data  (Attrition Bias) | Low | All clusters provided outcome data. Attrition as low as 60%. However, sensitivity analyses with multiple imputation showed similar findings. |
| Measurement of the Outcome (Detection Bias) | Some concerns | "Because of the nature of this study, only students, neither research assistants nor school administrators, were blind to intervention allocation". However, the study does not elaborate on how successful the blinding was. |
| Selection of the Reported Result (Reporting Bias) | Some concerns | Does not reference a protocol. |

## Hamilton et al. 2004

| Randomisation Process  (Selection Bias) | Some concerns | No information on how random allocation sequence was generated. |
| --- | --- | --- |
| Timing of identification or recruitment of participants (Selection Bias) | Low | Schools allocated to intervention and control conditions at the start of the study. |
| Deviations from intended interventions  (Performance Bias) | Some concerns | Does not specify if participants aware of trial status. Control group exposed to smoking curriculum due to governmental initiative; however, this is unlikely to have occurred because of the trial context. Does not specify if intention-to-treat analysis was used. |
| Missing Outcome Data  (Attrition Bias) | High | Large attrition rates, which increased with each post-test. Students who dropped out were more likely to smoke at baseline. Evidence of differential attrition between the trial groups. |
| Measurement of the Outcome (Detection Bias) | High | Not indicated if outcome assessors (participants) aware they are in a trial and not feasible to blind outcome assessors (participants) to intervention. |
| Selection of the Reported Result (Reporting Bias) | Some concerns | Does not reference a protocol. |

## Larsen et al. 2023

| Randomisation Process  (Selection Bias) | Low | "Representatives from Oxford Research who had no prior knowledge of the recruited schools allocated schools to a computer-generated randomisation list." |
| --- | --- | --- |
| Timing of identification or recruitment of participants (Selection Bias) | Low | Schools allocation to intervention and control conditions at the beginning of the study. |
| Deviations from intended interventions  (Performance Bias) | Some concerns | Does not specify if participants aware of trial status. Participants were unblinded to the intervention. Does not specify if deviations to trial context. Intention-to-treat analysis. |
| Missing Outcome Data  (Attrition Bias) | Low | "Low drop-out rates (8% in control and 5.4% in intervention group)." |
| Measurement of the Outcome (Detection Bias) | High | Not indicated if outcome assessors (participants) aware they are in a trial and not feasible to blind outcome assessors (participants) to intervention. |
| Selection of the Reported Result (Reporting Bias) | Some concerns | Protocol retrospectively registered, and some outcomes detailed in the protocol not presented in the paper (e.g. autonomy, school satisfaction). |

## Stevens et al. 2000

| Randomisation Process  (Selection Bias) | Some concerns | No details on how random allocation sequence was generated. |
| --- | --- | --- |
| Timing of identification or recruitment of participants (Selection Bias) | Low | Schools were allocated to intervention and control conditions at the beginning of the study. |
| Deviations from intended interventions  (Performance Bias) | Some concerns | Does not state if participants aware they are in a trial. Not feasible to blind participants to this type of intervention. Does not specifically address any deviations from intended intervention. Does not indicate if intention-to-treat analysis used. |
| Missing Outcome Data  (Attrition Bias) | High | All clusters responded with outcome data.  26% attrition. Does not provide multiple imputation, and sensitivity analyses seem to indicate differential results in dropout sample. |
| Measurement of the Outcome (Detection Bias) | High | Not indicated if outcome assessors (participants) aware they are in a trial and not feasible to blind outcome assessors (participants) to intervention. |
| Selection of the Reported Result (Reporting Bias) | Some concerns | No protocol referenced. Paragraphs often only describe significant results but all results accessible in tables. |

## Rahman et al. 1998

| Randomisation Process  (Selection Bias) | Low | "Schools were stratified and then one school for boys and one school for girls were randomly allocated to the intervention and control conditions. The school’s names were drawn by a colleague who was not involved in the research”. |
| --- | --- | --- |
| Timing of identification or recruitment of participants (Selection Bias) | Low | Schools were allocated to intervention and control arms at the beginning of the study. |
| Deviations from intended interventions (Performance Bias) | Some concerns | Does not detail whether deviations from intended design occurred; however, flow diagram indicates that ITT analysis was used." |
| Missing Outcome Data  (Attrition Bias) | Low | No participants lost-to-follow-up |
| Measurement of the Outcome (Detection Bias) | High | Not indicated if outcome assessors (participants) aware they are in a trial and not feasible to blind outcome assessors (participants) to intervention. |
| Selection of the Reported Result (Reporting Bias) | Some concerns | No protocol available. |

## Skarstrand et al. 2013

| Randomisation Process  (Selection Bias) | Some concerns | No information on how random allocation sequence was generated. |
| --- | --- | --- |
| Timing of identification or recruitment of participants (Selection Bias) | Low | Schools allocated to intervention and control conditions at the beginning of the study. |
| Deviations from intended interventions  (Performance Bias) | Some concerns | "One intervention school and two control schools declined participation after randomization, leaving 10 intervention schools and 9 control schools. Five of the schools wanted to include more than one class per school in the study; hence, the final population consisted of students from 15 classes in the intervention  group and 11 classes in the control group.” Relatively balanced between the two groups.  Intention-to-treat analysis was used. |
| Missing Outcome Data  (Attrition Bias) | Low | "The maximum number of missing values of the outcomes varied between 18% at baseline and 26% at the last follow-up. Missing data at the last follow-up was quantitatively equal between intervention and control groups. As expected, the students with missing values of the outcomes at the last follow-up were significantly more likely to be male and to have higher baseline prevalence of drunkenness compared with students who had answered the questionnaire. The interaction terms between intervention condition and baseline characteristics of the students were not statistically significant for any variable (data not shown), indicating that patterns of missingness  were similar across experimental conditions. The sensitivity analysis and all analyses performed on imputed data confirmed results of the available case analysis, indicating no effectiveness of the prevention programme (data not shown)." |
| Measurement of the Outcome (Detection Bias) | High | Not indicated if outcome assessors (participants) aware they are in a trial and not feasible to blind outcome assessors (participants) to intervention. |
| Selection of the Reported Result (Reporting Bias) | Some concerns | No protocol available. |

## Wolfe et al. 2009

| Randomisation Process  (Selection Bias) | Low | "Schools were randomly assigned by strata to intervention or control on the basis of a coin toss" |
| --- | --- | --- |
| Timing of identification or recruitment of participants (Selection Bias) | Low | Schools were allocated to intervention and control conditions at the beginning of the study. |
| Deviations from intended interventions  (Performance Bias) | Low | "Students were masked to condition in that they were aware only that they were receiving Health class. Students were masked to the objectives of the study, and they completed additional health-related survey questions (e.g. smoking, diet and exercise, and peer and family relationships) to mask the primary outcome.” Intention-to-treat analysis used. |
| Missing Outcome Data  (Attrition Bias) | Low | Low rates of attrition overall, with non‐differential attrition between trial groups. No differences between trial groups with regard to the primary outcome of dating violence. |
| Measurement of the Outcome (Detection Bias) | Some concerns | "Students were masked to condition in that they were aware only that they were receiving a health class." Teachers, however, were aware of school assignment. Does not elaborate on how successful the blinding process was. |
| Selection of the Reported Result (Reporting Bias) | Some concerns | No protocol available |
