# Supplementary material for: Systematic Review and Meta-analysis of the Effectiveness of Whole-school Interventions Promoting Mental Health and Preventing Risk Behaviours in Adolescence
Source: J Youth Adolesc. 2025 Jan 27;54(2):271–89. doi: 10.1007/s10964-025-02135-6 (PMC11807013; doi:10.1007/s10964-025-02135-6)
Supplement: Supplementary file 3 — Supplementary Materials 3_Individual Forrest Plots [file 10964_2025_2135_MOESM3_ESM.docx]

**Systematic Review and Meta-Analysis of the Effectiveness of Whole-School Interventions Promoting Mental Health and Preventing Risk Behaviours in Adolescence**

SUPPLEMENTARY MATERIALS 3:
INDIVIDUAL FOREST PLOTS

# ANY SMOKING

# REGULAR SMOKING

# ANY ALCOHOL USE

# REGULAR ALCOHOL USE

# OTHER SUBSTANCE USE

#

# ANXIETY

# DEPRESSION

#

# PSYCHOLOGICAL SYMPTOMS

#

# IN-PERSON BULLYING

#

# CYBER-BULLYING

# IN-PERSON AGGRESSION

# CYBER-AGGRESSION

# EMOTIONAL AND PSYCHOLOGICAL WELLBEING

SOCIAL WELLBEING
