# Supplementary material for: Systematic Review and Meta-analysis of the Effectiveness of Whole-school Interventions Promoting Mental Health and Preventing Risk Behaviours in Adolescence
Source: J Youth Adolesc. 2025 Jan 27;54(2):271–89. doi: 10.1007/s10964-025-02135-6 (PMC11807013; doi:10.1007/s10964-025-02135-6)
Supplement: Supplementary file 4 — Supplementary Materials 4_Baseline Outcomes [file 10964_2025_2135_MOESM4_ESM.docx]

**Systematic Review and Meta-Analysis of the Effectiveness of Whole-School Interventions Promoting Mental Health and Preventing Risk Behaviours in Adolescence**

SUPPLEMENTARY MATERIALS 4:
BASELINE RATES OF OUTCOMES

| **Study** | **Baseline Rates of Outcomes** |
| --- | --- |
| Allara 2019 | Current smoker n (%): Intervention 291 (22.47); Control 283 (19.38); Total 574 (20.83)  Frequent smoker n (%): Intervention 105 (8.11); Control 136 (9.32); Total 241 (8.75)  Current alcohol intoxication n (%): Intervention 143 (11.05); Control 170 (11.77); Total 313 (11.43)  Frequent alcohol intoxication n (%): Intervention 54 (4.17); Control 57 (3.95); Total 111 (4.05) |
| Andersen 2019 | Current smoker n (%): Intervention 129 (5.6); Control 131 (7.6) |
| Bond 2004 | Control, Intervention, Total n (%)  Bullied n (%): Control 724 (53.9); Intervention 687 (51.5); Total 1411 (52.7)  Depressive symptoms n (%): Control 253 (18.8); Intervention 217 (16.3); Total 470 (17.6)  Alcohol drinker n (%): Control 432 (32.2); Intervention 397 (29.7); Total 829 (31.0)  Regular drinker n (%): Control 75 (5.6); Intervention 62 (4.6); Total 137 (5.1)  Binge drinking n (%): Control 174 (13.0); Intervention 167 (12.5); Total 341 (12.7)  Smoker (any) n (%): Control 214 (15.9); Intervention 201 (15.1); Total 415 (15.5)  Regular smoker n (%): Control 38 (2.8); Intervention 25 (1.9); Total 63 (2.4)  Marijuana not used n (%): Control 1112 (82.8); Intervention 1205 (90.3),; Total 2317 (86.5) |
| Bonell et al., 2018; Bonell et al., 2019; Bonell et al., 2020; Melendez-Torres et al., 2021; Melendez-Torres et al., 2022; Warren et al., 2019 | Control, intervention, overall mean (SD).  Bullying victimisation mean (SD): Control 0·51 (0·63); Intervention 0·48 (0·60); Total 0·49 (0·61)  Aggressive behaviours mean (SD): Control 2·92 (4·84); Intervention 2·72 (4·77); Total 2·82 (4·81)  Psychological problems mean (SD): Control 11·00 (5·99); Intervention 10·70 (5·76); Total 10·85 (5·88)  Wellbeing mean (SD): Control 24·11 (5·91); Intervention 24·33 (5·91); Total 24·21 (5·91)  Life satisfaction mean (SD): Control 80·39 (14·31); Intervention 80·98 (14·08); Total 80·68 (14·20) Ever smoked n (%): Control 189 (5·78); Intervention 151 (4·72); Total 340 (5·25).  Ever consumed alcohol n (%): Control 487 (15·04); Intervention 393 (12·37); Total 880 (13·72).  Cyberbullying perpetration n (%): Control 290 (8.9); Intervention 279 (8.6); Total 569 (8.7)  Cyberbullying victimisation n (%): Control 522 (16.0); Intervention 467 (14.5); Total 989 (15.3)  E-cigarette use n (%): Control 187 (5.8); Intervention 131 (4.2); Total 318 (5.0). |
| Bonnesen et al., 2023; Bonnesen et al., 2020 | Perceived stress mean (SD): Intervention 13.8 (6.5); Control 14.2 (6.4) |
| Cross et al., 2016 | Cybervictimisation mean (SD): Intervention 0.10 (0.26), Control 0.08 (0.25), Total 0.09 (0.25)  Cyber-perpetration mean (SD): Intervention 0.03 (0.17), Control 0.02 (0.12), Total 0.03 (0.15)  Not involved in cyber-victimisation at baseline %: Intervention 70; Control 73; Total 72  Not involved in cyber-perpetration at baseline %: Intervention 89; Control 91; Total 90 |
| Cross et al., 2018 | Victimisation mean (SD), % zero: Intervention 0.223 (0.266), 34.3; Control 0.190 (0.2506), 39.9; Total 0.207 (0.259), 37.1  Perpetration mean (SD), % zero: Intervention 0.106 (0.167), 55.4; Control 0.088 (0.162), 60.9;  Total 0.097 (0.165), 58.1  Depression mean (SD), % zero: Intervention: 0.254 (0.333), 45.3; Control 0.242 (0.334), 49.7;  Total 0.248 (0.333), 47.4  Anxiety mean (SD), % zero: Intervention 0.247 (0.303), 39.1; Control 0.221 (0.293), 44.4;  Total 0.234 (0.298), 41.7 |
| de Vries et al., 2006 (Denmark); de Vries et al., 2003 (Denmark) | Weekend alcohol consumption %: Intervention 17.3; Control 16.4  Fewer students from control schools reported drinking alcohol during the week compared to those from experimental schools [8.2 versus 8.8%, P < 0. 001] |
| de Vries et al., 2006 (Finland); de Vries et al., 2003 (Finland); Vartiainen et al., 2007 |  |
| Dray et al., 2017; Hodder et al., 2017; Hodder et al., 2018 | Psychological problems mean (SD): Control 2.4 (6.3); Intervention 13.5 (6.6)  Tobacco use (ever) mean (SD): Control 124 (10.5); Intervention 221 (11.7)  Tobacco use (recent) mean (SD): Control 21 (1.8); Intervention 49 (2.6)  Alcohol use (ever) mean (SD): Control 316 (26.7); Intervention 615 (32.5)  Alcohol use (recent) mean (SD): Control 53 (4.5); Intervention 121 (6.4)  Alcohol use (risky) mean (SD): Control 50 (4.2); Intervention 111 (5.9)  Marijuana use mean (SD): Control 12 (1.0); Intervention 34 (1.8) |
| Foshee et al., 1998; Foshee et al., 2004; Foshee et al., 2005; Foshee et al., 2014 | Psychological abuse mean: Intervention 0.58; Control 0.58 Nonsexual violence mean: Intervention 0.26; Control 0.22 Violence in current relationship: Intervention 0.04; Control 0.03 |
| Gorini et al., 2014; Carreras et al., 2016 | Past 30-day cigarette use %: Control 21.4; Intervention 17  ≥ 20 days of cigarette smoking in past 30 days %: Control 5.2; Intervention 2.7 |
| Hamilton et al., 2005; Hamilton et al., 2007 | Never smoker %: Intervention 50.2; Control 45.9  30-day prevalence %: Intervention 19.4; Control 22.0  Regular smoker %: Intervention 7.5; Control 10.2 |
| Hunt, 2007 | Experience being bullied mean (SD): Intervention 5.14 (1.21); Control 5.29 (1.05)  Ability to join in bullying mean (SD): Intervention 4.34 (1.52); Control 4.34 (1.59) |
| Johnson et al., 2017 | Depression mean (SD): Intervention 0.77 (0.65); 0.74 (0.71)  Anxiety mean (SD): Intervention 0.87 (0.57); Control 0.86 (0.63)  Wellbeing mean (SD): Intervention 3.46 (0.66); Control 3.53 (0.70) |
| Kärnä et al., 2013 | Self-reported victimisation mean (SD): Control 0.10 (0.30); Intervention 0.09 (0.29)  Self-reported bullying mean (SD): Control 0.08 (0.26); Intervention 0.07 (0.25) |
| Larsen et al., 2023; Larsen et al., 2021 | Mental health problems mean (SD): Control 1.84 (0.82); Intervention 1.75 (0.79) |
| Malmberg et al., 2014; Malmberg et al., 2015 | Alcohol lifetime %: Control 25.4; Intervention 32.2  Alcohol binge %: Control 94.1; Intervention 90.5  Tobacco lifetime %: Control 17.3; Intervention 26.  Marijuana lifetime %: Control 1.3; Intervention 2.8 |
| Perry et al., 2003; Komro et al., 2004; Bosma et al., 2005 | Alcohol past month mean (SD) in males: Control 1.11 (0.02); Intervention 1.09 (0.02)  Alcohol ever drunk mean (SD) in males: Control 1.09 (0.02); Intervention 1.07 (0.02).  Tobacco current smoker mean (SD) in males: Control 1.29 (0.06); Intervention 1.31 (0.06)  Marijuana behaviour and intentions mean (SD) in males: Control 6.47 (0.08); Intervention 6.49 (0.08)  Violent behaviour and intentions mean (SD) in males: Control 7.92 (0.17); Intervention 7.82 (0.16) Physical victimisation mean (SD) in males: Control 4.19 (0.15); Intervention 4.16 (0.15)  Alcohol past month mean (SD) in females: Control 1.08 (0.02); Intervention 1.08 (0.02)  Alcohol ever drunk mean (SD) in females: Control 1.07 (0.02); Intervention 1.07 (0.02)  Tobacco current smoker mean (SD) in females: Control 1.31 (0.07); Intervention 1.43 (0.06)  Marijuana behaviour & intentions mean (SD) in females: Control 6.32 (0.10); Intervention 6.43 (0.09)  Violent behaviour and intentions mean (SD) in females: Control 6.66 (0.16); Intervention 6.67 (0.15) Physical victimisation mean (SD) in females: Control 3.44 (0.10); Intervention 3.37 (0.10) |
| Perry et al., 2009; Bate et al., 2009 | Cigarette smoking % (95% CI): Intervention 0.00 (−0.77, 0.77); Control 0.43 (−0.34, 1.20) |
| Rahman et al., 1998 | Mental health literacy total score: Intervention 6.4; Control 7.2 |
| Sawyer et al., 2010b; Sawyer et al., 2010a; Spence et al., 2014 | Depressive symptoms mean (SD): Total 13.5 (10.9)  Optimistic thinking style mean (SD): Total 24.2 (7.6)  Positive coping mean (SD): Total 25.7 (9.6)  Adolescent school climate mean (SD): Total 60.1 (9.9) |
| Schofield et al., 2003 | Total 19% had smoked in the last month  Total 8% had smoked in the last week (3.9% intervention and 4.1% control) |
| Shinde et al., 2020; Shinde et al., 2018; Singla et al., 2021 | School climate score mean (SD): Intervention 17·87 (4·1); Control 17·93 (4·0)  Depressive symptoms mean (SD): Intervention 6·61 (5·3); Control 6·40 (5·2)  Violence (victimisation) in the past 12 months n (%): Intervention 723 (16); Control 772 (17)  Violence (perpetration) in the past 12 months n (%): Intervention 579 (13); Control 575 (13)  Tobacco smoking in the past 12 months n (%): Intervention 140 (3); Control156 (4)  Alcohol drinking in the past 12 months n (%): Intervention 158 (4); Control 151 (3)  Other substance use in the past 12 months n (%): Intervention 275 (6); Control 232 (5)  Suicide attempt in the past 12 months n (%): Intervention 81 (2); Control 107 (2) |
| Skärstrand et al., 2014 | Baseline smokers %: Intervention 1; Control 3.2  Drunkenness lifetime %: Intervention 7.5; Control 7.1  Illicit substance use %: Intervention 0.6; Control 1 |
| Stevens et al., 2000; Stevens et al., 2001 | Being bullied mean (SD): Intervention 1.02 (0.11); Control 1.03 (0.12)  Bullying others mean (SD): Intervention 0.99 (0.09); Control 1.02 (0.12) |
| Wen et al., 2010; Wen et al., 2007 | Ever smoking %: Total 19.9 (29.1% of males; 8.8% of females)  Regular smoking %: Total 4.5 (7.9% of males; 0.7% of females) |
| Wolfe et al., 2009 | Physical dating violence in past year %: Control 1.1; Intervention 1  Physical peer violence in previous 3 months %: Control 22.9; Intervention 27  Alcohol problem use %: Control 18; Intervention 17  Drug problem use %: Control 9.9; Intervention 13 |
